# Enhanced Activities of OCT4 and SOX2 Promote Epigenetic Reprogramming by Shortening G1 Phase

**DOI:** 10.1002/advs.202415528

**Published:** 2025-05-31

**Authors:** Lin Guo, Jiechun Lin, Qiwen Ren, Hao Sun, Yanhua Wu, Haofei Ge, Xiaolan Wu, Lihui Lin, Lining Liang, Changpeng Li, He Liu, Yuangbang Mai, Shilong Chu, Jiadong Liu, Jing Liu, Jiekai Chen, Duanqing Pei, Hui Zheng

**Affiliations:** ^1^ Guangdong Provincial Key Laboratory of Stem Cell and Regenerative Medicine Guangdong‐Hong Kong Joint Laboratory for Stem Cell and Regenerative Medicine GIBH‐CUHK Joint Research Laboratory on Stem Cell and Regenerative Medicine Guangzhou Institutes of Biomedicine and Health Chinese Academy of Sciences Guangzhou 510530 China; ^2^ Joint School of Life Sciences Guangzhou Medical University Guangzhou 511436 China; ^3^ University of Chinese Academy of Sciences Beijing 100049 China; ^4^ Centre for Regenerative Medicine and Health Hong Kong Institute of Science & Innovation Chinese Academy of Sciences Hong Kong SAR 999077 China; ^5^ Laboratory of Cell Fate Control School of Life Sciences Westlake University Hangzhou 310024 China

**Keywords:** cell cycle, histone methylation, OvSvK, reprogramming, shortened G1 phase

## Abstract

To investigate the interplay among OCT4, SOX2, and KLF4, the VP16 activation domain is fused to one or multiple of these factors during reprogramming. Fusion of VP16 with OCT4 and SOX2 (OvSvK) significantly increases the efficiency of induced pluripotent stem cell (iPSC) generation compared to other combinations. The enhanced activities of OCT4 and SOX2 directly facilitated reprogramming by activating downstream targets, some of which are involved in cell cycle regulation. This leads to a shortened G1 phase and a shift in cell cycle dynamics toward an iPSCs‐like state. Further analysis reveals that these cell cycle alterations reduced H3K27me3 levels on specific genes, thereby promoting reprogramming. Consistently, knockdown of *Ccnd1* and *Cdkn2a* (si*Ccnd1*, si*Cdkn2a*) as well as overexpression of *Ccne1* effectively shortened the G1 phase and enhanced reprogramming efficiency. These findings highlight the role of cell cycle modulation in epigenetic remodeling and provide mechanistic insights for optimization during somatic cell reprogramming.

## Graphical abstract

Fusing the VP16 domain to OCT4 and SOX2 (OvSvK) enhances iPSC generation by activating downstream targets, including those regulating the cell cycle. This accelerates reprogramming by shortening the G1 phase and reducing H3K27me3 levels. Modulating *Ccnd1*, *Cdkn2a*, and *Ccne1* improves efficiency, linking cell cycle to epigenetic remodeling.

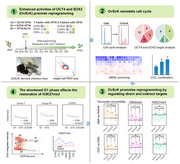

## 1 Introduction

The ability of transcriptional factors to drive cell fate conversion have been demonstrated during the generation of induced pluripotent stem cells (iPSCs).^[^ ^1^ ^]^ The transdifferentiation of mouse embryonic fibroblasts (MEFs) into functional neurons further extends the scope of applying exogenous transcription factors.^[^ ^2^ ^]^ Among these, OCT4 and SOX2 are recognized as pioneer transcription factors that bind partial motifs on the surface of nucleosomes and initiate the reprogramming gene network.^[^ ^3^, ^4^ ^]^ The binding of OCT4, SOX2, and KLF4 represses somatic enhancers while activating pluripotency enhancers, thereby altering the epigenetic landscapes of the cells.^[^ ^5^ ^]^ However, these transcription factors exhibit a dual effect. For instance, KLF4 can drive cells toward an unexpected non‐reprogramming path.^[^ ^6^ ^]^ Furthermore, when the exogenous factors were introduced in a specific sequence (*Oct4* and *Klf4* first, then *c‐Myc*, and finally *Sox2*), a temporal epithelial‐mesenchymal transition (EMT) preceded the conventional mesenchymal‐ epithelial transition (MET) and facilitated the reprogramming.^[^ ^7^ ^]^

Therefore, it is reasonable to infer that varying activation levels of these factors might lead to distinct reprogramming. The herpes simplex virus‐encoded protein VP16 is a potent transcriptional activator with a strong activation domain.^[^ ^8^ ^]^ Previous studies have shown that fusing the VP16 domain with OCT4, SOX2 or NANOG enhances reprogramming efficiency.^[^ ^9^ ^]^ In this study, to modulate the transcriptional activity of these factors, the VP16 transcription activation domain was fused to one or two of OCT4, SOX2, and KLF4 (OvSK, OSvK, OSKv, OvSvK, OvSKv, and OSvKv). Intriguingly, we found the OvSvK combination significantly improved reprogramming and altered the cell cycle dynamics of somatic cells to an ESC‐like state. Given that cell proliferation is critical for various types of cell fate conversions, including somatic cell reprogramming,^[^ ^10^, ^11^, ^12^, ^13^ ^]^ and that a key distinction between ESCs and somatic cells, such as MEFs, is a shortened G1 phase with an increased S phase percentage, we hypothesized that OvSvK promotes reprogramming by remodeling the cell cycle, at least partially.

Additionally, epigenetic reprogramming is essential for somatic cell reprogramming.^[^ ^14^ ^]^ By modulating the epigenetic modification state, the efficiency of reprogramming and other cell fate changes could be regulated.^[^ ^15^, ^16^, ^17^ ^]^ Since epigenetic modifications, such as DNA methylation and histone methylation, are replicated and inherited by daughter cells during cell division, a connection between cell division and epigenetic reprogramming is plausible. Recent studies indicate DNA methylation inheritance is incomplete during S phase and requires further reinforcement during G2/M phase or even the subsequent G1 phase.^[^ ^18^, ^19^ ^]^ Studies on Hela cells have revealed distinct restoration kinetics of H3K4me3 and H4K27me3 throughout the cell cycle, suggesting that the inheritance of histone modification is more complex.^[^ ^20^, ^21^ ^]^ However, the mechanisms underlying histone inheritance and its regulation during cell fate transitions remain poorly understood.

To this end, we focused on the shortened G1 phase of cells during OvSvK reprogramming and examined its relationship with cell cycle remodeling and epigenetic modification. Through multi‐omics analyses, we found that the shortened G1 phase induced histone modification changes that facilitated reprogramming. This novel finding is crucial for understanding reprogramming mechanisms and has broad implications for the study of cell fate transitions.

## 2 Results

### 2.1 OvSvK Optimizes Somatic Cell Reprogramming

To investigate the interplay among OCT4, SOX2, and KLF4, we first assessed their contributions to reprogramming barriers using RNA‐seq after introducing one or two of these factors during reprogramming (Figure S1a, Supporting Information). Reprogramming barriers were identified by analyzing transcriptomic data from MEFs, iPSCs, ESCs, and pre‐iPSCs, an intermediate and barrier status during reprogramming (GSE14012 and GSE10871).^[^ ^22^, ^23^ ^]^ Genes whose expression levels were significantly higher in pre‐iPSCs (log_2_ difference >1) than in MEFs, iPSCs, and ESCs were classified as over‐upregulated barriers and were predominantly targeted by KLF4 rather than OCT4 and SOX2 (Figure S1b, Supporting Information). Conversely, genes whose expression in iPSCs and ESCs than in MEFs and pre‐iPSCs were classified as insufficiently upregulated barriers, primarily regulated by OCT4 and SOX2 rather than KLF4 (Figure S1c, Supporting Information). Similar patterns were observed for over‐downregulated and insufficiently‐downregulated barriers (Figure S1d,e, Supporting Information). These findings suggest that enhancing the activities of OCT4 and SOX2 may improve reprogramming efficiency.

**Figure 1:**
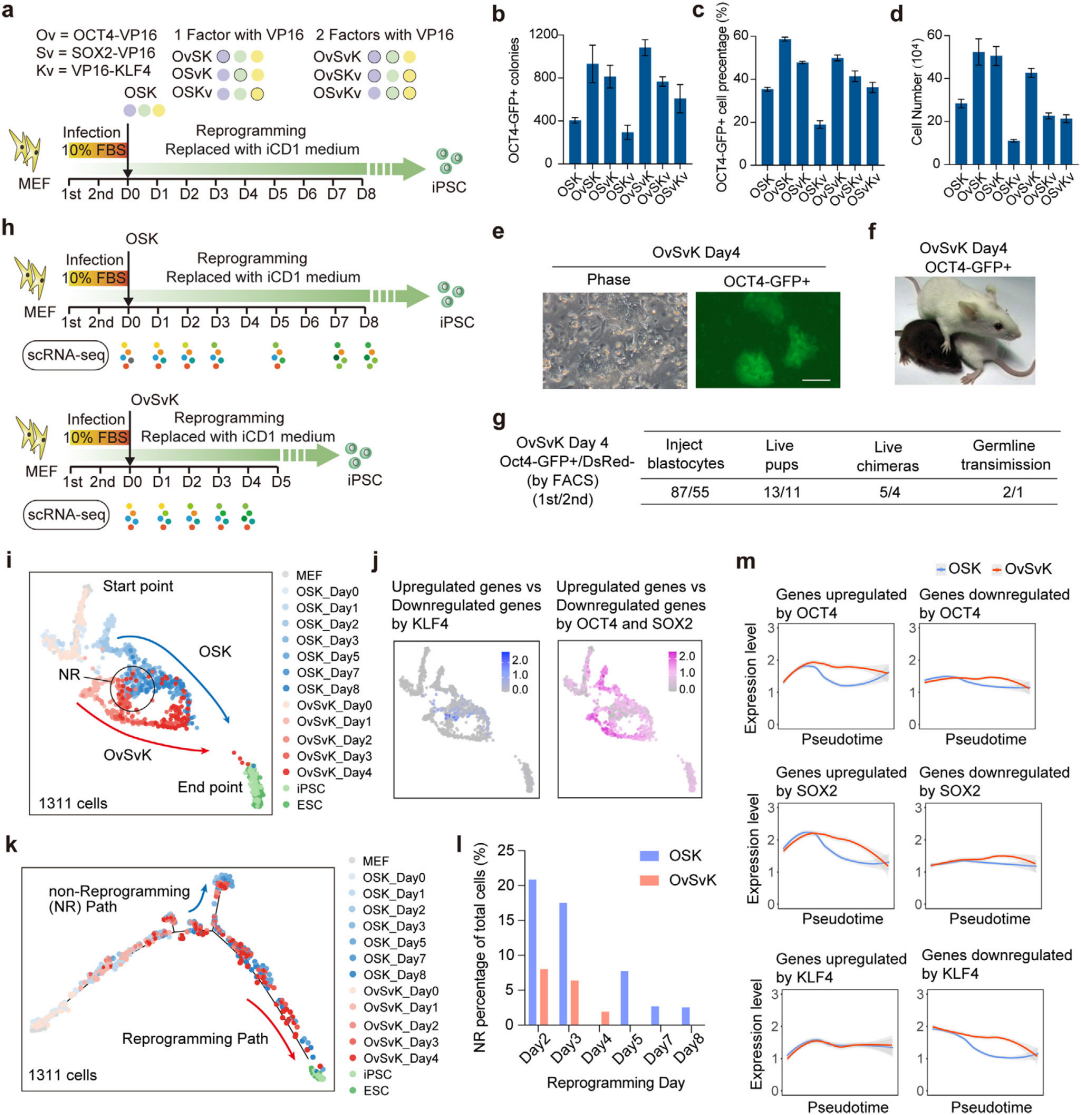
VP16‐fused OCT4 and SOX2 accelerate reprogramming. a) Schematic diagram of reprogramming with different combinations of factors. b) OCT4‐GFP^+^ colonies were counted at day 5 post‐infection (*n* = 4, mean ± SD). c) OCT4‐GFP^+^ cells were detected at day 5 post‐infection by FACS (*n* = 4, mean±SD). d) Cell proliferation analysis with different factor combinations at day 5 post‐infection (*n* = 4, mean ± SD). e–g) OvSvK‐derived iPSCs were OCT4‐GFP^+^ (e, scale bar, 100 µm) and capable of deriving chimera mice with germline transmission (f,g). h) Schematic diagram of single‐cell RNA‐sequencing. i) A total of 1311 cells were displayed. The red and blue lines show the reprogramming paths of OvSvK and OSK, respectively. Cells in the circle indicate the non‐reprogramming (NR) cells. j) The abilities of OCT4/SOX2 and KLF4 to regulate downstream targets (the expression of upregulated genes minus those of downregulated genes) were plotted. k) Pseudotime analysis of the two reprogramming systems. l) Percentage of NR cells at each time point. m) The expression of genes that were upregulated or downregulated by OCT4, SOX2, and KLF4 was analyzed along the pseudotime course. The upregulation induced by OCT4 and SOX2 was enhanced, while the downregulation induced by KLF4 was impaired.

To validate this hypothesis, we fused the VP16 transcription activation domain individually to OCT4, SOX2, and KLF4 (Figure S2a, Supporting Information). Various combinations of intact and fused factors were then introduced into MEFs simultaneously (**Figure** **1**a). Among these new reprogramming systems, OvSvK exhibited the highest efficiency to generate OCT4‐GFP^+^ colonies, producing ≈2.5 times more colonies than the conventional OSK system (Figure 1b,e). We analyzed the proportion of OCT4‐GFP^+^ cells using flow cytometry, which exhibited a consistent result with that of OCT4‐GFP^+^ colonies (Figure 1c). Cell counting results indicated that Ov and Sv promoted the proliferation of reprogrammed cells, whereas Kv inhibited cell proliferation (Figure 1d). To determine whether the lack of improvement in OSKv reprogramming efficiency was due to the N‐terminal placement of VP16‐Flag in KLF4, we constructed a new vector with VP16‐Flag at the C‐terminal (pMXs‐Kvc) (Figure S2a, Supporting Information). The results showed that C‐terminal VP16‐Flag also inhibited reprogramming efficiency and cell proliferation, similar to N‐terminal fusion (Figure S2b–d, Supporting Information). Thus, the lack of significant improvement in OSKv reprogramming efficiency is not attributable to the N‐terminal versus C‐terminal placement of VP16‐Flag.

We analyzed the protein levels of OCT4, SOX2, KLF4, as well as Ov and Sv. The results indicated that Ov/Sv expression was slightly lower than that of O/S, suggesting that enhanced reprogramming efficiency of OvSvK was not attributable to increased protein levels (Figure S2e, Supporting Information). We further performed a dual‐luciferase reporter assay to compare the transcriptional activities of OCT4, SOX2, and their VP16‐fused counterparts. The results demonstrated that the transcriptional activity of the factors was significantly enhanced upon fusion with VP16 (Figure S2f, Supporting Information). Since exogenous retroviral vectors are silenced in reprogrammed cells, we used DsRed as an indicator of viral silencing. OCT4‐GFP^+^/DsRed^−^ cells, isolated as early as day 4 during OvSvK reprogramming, were directly injected into the mouse blastocytes and demonstrated the ability to contribute to chimera formation and germline transmission (Figure 1f,g). Additionally, when MEFs at different passages were reprogrammed using OSK and OvSvK, we observed that increasing passage number were associated with signs of cellular senescence (Figure S3c, Supporting Information). Although OvSvK remained effective in promoting reprogramming, both overall reprogramming efficiency and proliferative capacity declined as passage number increased (Figure S3a,b, Supporting Information). These findings indicate that the enhanced activities of OCT4 and SOX2 accelerate reprogramming.

### 2.2 scRNA‐seq analysis of the reprogramming process mediated by OSK and OvSvK

Given that OvSvK generated chimera‐competent iPSCs by day 4 of reprogramming, we collected cells at days 0, 1, 2, 3, and 4 of OvSvK reprogramming for single‐cell RNA‐sequencing (scRNA‐seq). These data were then compared with previously obtained OSK reprogramming datasets (day 0, 1, 2, 3, 5, 7, and 8)^[^ ^6^ ^]^ (Figure 1h). First, we examined the expression levels of various transcription factors in OSK and OvSvK, confirming that all related TFs were expressed in the two systems (Figure S4a, Supporting Information). We then compared the expression levels of upregulated and downregulated genes during reprogramming in both systems using real‐time and pseudotime analyses. The results indicated that by day 4, OvSvK exhibited a gene expression profile comparable to that of OSK at day 8 (Figure S4b,c, Supporting Information). A similar trend was observed in the expression of pluripotency‐associated genes (Figure S4d, Supporting Information). These findings suggested that day 4 can be considered as the endpoint of OvSvK reprogramming.

The analysis revealed that while the OSK and OvSvK reprogramming systems re‐established pluripotency through distinct routes, they shared a similar non‐reprogramming (NR) route (Figure 1i,k). To identify differentially regulated genes, we compared the transcriptomes of MEFs and MEFs with only KLF4 over‐expression. Upregulated and downregulated genes were identified, and their expression changes were summarized and visualized in Figure 1j. Notably, these genes exhibited greater expression changes in cells following the NR route. A similar analysis was conducted for OCT4 and SOX2, revealing that genes regulated by these factors underwent more substantial changes in cells undergoing reprogramming route (Figure 1j). This finding aligns with our previous observation that the exogenous KLF4 expression during reprogramming contributes to the NR route.^[^ ^6^ ^]^

Although the OSK and OvSvK reprogramming systems shared a similar NR route, a higher proportion of cells in the OSK system were classified into this NR route compared to the OvSvK system (Figure 1i). This suggests that enhanced transcriptional activities of OCT4 and SOX2 may disrupt the balance among OCT4, SOX2, and KLF4. After excluding the cells in the NR routes, we analyzed the regulatory effects of OCT4, SOX2, and KLF4 on their downstream targets along the pseudotime course. We observed enhanced transcriptional activities of OCT4 and SOX2, while KLF4 activity was suppressed during OvSvK reprogramming (Figure 1m). Therefore, enhancing the activities of OCT4 and SOX2 while suppressing KLF4 facilitated reprogramming, consistent with the previously reported negative role of KLF4 in this process.^[^ ^6^ ^]^

### 2.3 OvSvK Remodels Cell Cycle by Shortening G1 Phase

The average expression of all detected genes during OSK and OvSvK reprogramming was calculated, and a comparison of the overall transcriptomes between the two reprogramming systems identified 3,562 genes (set 1) with higher expression and 2,271 genes (set 2) with lower expression in OvSvK system. Most of these differences contributed to overcoming the previously mentioned reprogramming barriers (Figure S4e, Supporting Information). Additionally, OCT4 and SOX2 preferentially regulated the genes in set 1, and their enhanced activities likely led to the higher expression of these genes in OvSvK (Figure S4f, Supporting Information). KLF4 preferentially regulated the genes in set 2, and its suppressed activity may result in their lower expression in OvSvK (Figure S4g, Supporting Information).

**Figure 2:**
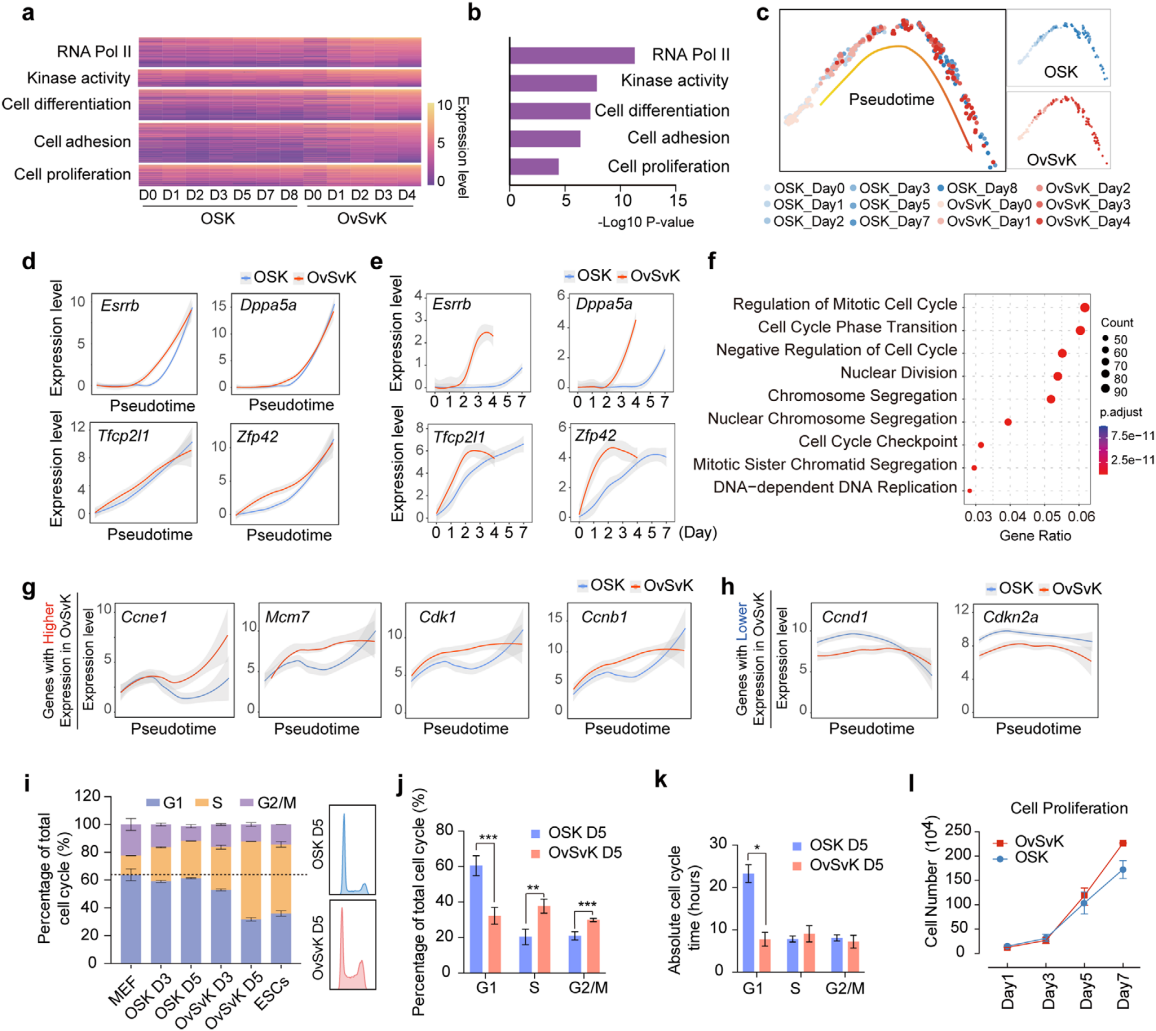
OvSvK reprogramming established an ESC‐like cell cycle character. a) Heatmap showed the results of the GO analysis of OvSvK and OSK reprogramming on different days. b) The enrichment scores of the six groups between OvSvK and OSK systems. c) Pseudotime analysis showed reprogramming cells without NR cells. d,e) Expression levels of pluripotent genes changed with pseudotime and real‐time analysis. f) GO analysis of the upregulated genes in OvSvK after pseudotime analysis. g,h) Expression level of cell cycle genes changed with pseudotime. i) Reprogrammed cells at different time points and ESCs were stained with propidium iodide and analyzed by FACS (*n* = 3, mean ± SD). The right panel showed FACS analysis of OSK and OvSvK on day 5. j) Comparison of cell cycle distribution (G1, S, and G2/M phases) between OvSvK and OSK (*n* = 4, mean±SD). ^**^ *p* < 0.01; ^***^ *p* < 0.001, Student's *t*‐test. k) Cell counting of reprogramming cells in OSK and OvSvK on day 4 and day 5, followed by the calculation of cell doubling time. Absolute proliferation time was determined based on the cell cycle distribution shown in Figure 2j (*n* = 2, mean ± SD). ^*^ *p* < 0.05, Student's *t*‐test. l) Cell proliferation of OSK and OvSvK reprogramming at different days (*n* = 3, mean±SD).

We then focused on the genes with higher expression in OvSvK (set 1) to investigate how the enhanced activities of OCT4 and SOX2 optimize reprogramming. Gene Ontology (GO) analysis revealed that set 1 genes were associated with RNA polymerase II activity, kinase activity, cell differentiation, cell adhesion, and cell proliferation (**Figure** **2**a,b). The expression of set 1 genes were gradually increased in both OSK and OvSvK systems, but exhibited a more rapid and pronounced upregulation in OvSvK (Figure 2a).

To eliminate the influences of the accelerated re‐establishment of pluripotency in the OvSvK system and to investigate the underlying mechanisms driving the promoted reprogramming, we compared the transcriptomes of the two reprogramming systems along the pseudotime course, in addition to analyzing the average gene expression over real time (Figure 2c). Using this approach, the upregulation of pluripotency‐related genes became similar in the two reprogramming systems at pseudotime (Figure 2d). However, genes such as *Esrrb* and *Dppa5a* which are upregulated during the late reprogramming stage in OSK showed significant difference between OSK and OvSvK at real time (Figure 2e). Similarly, genes such as *Tfcp2l1* and *Zfp42*, which are upregulated during the early reprogramming stage in OSK, showed rapid upregulation in OvSvK (Figure 2e). These changes, introduced by the utilization of new methods, were less pronounced for genes associated with pluripotency. GO analysis revealed that these 1,523 genes upregulated in OvSvK in pesudotime were strongly associated with the cell cycle (Figure 2f). Specifically, genes such as *Ccne1*, *Mcm7, Cdk1*, and *Ccnb1* were activated earlier and reached higher expression levels during OvSvK reprogramming (Figure 2g). Additionally, although cell cycle‐related genes were not enriched among the 732 genes with lower expression in the OvSvK system, several key regulators of the cell cycle, including *Ccnd1* and *Cdkn2a*, exhibited reduced expression in the OvSvK system (Figure 2h).

We then analyzed cell cycle dynamics on day 5 of reprogramming and observed that the G1 phase was shorter in the OvSvK system than that in the OSK system (Figure 2i,j). Further studies confirmed that the cell cycle dynamics of MEFs were gradually remodeled to resemble those of ESCs, with this remodeling occurring more rapidly in OvSvK reprogramming (Figure 2i). Significant differences in various cell cycle phases were detected between OSK and OvSvK on day 5 of reprogramming (Figure 2j). Additionally, we calculated the cell doubling time between days 4 and 5 and determined the absolute proliferation time based on the cell cycle distribution on day 5. The results revealed that OvSvK exhibited a significant shorter G1 phase compared to OSK, whereas the absolute durations of the S and G2/M phases remained largely comparable between the two conditions (Figure 2k). Meanwhile, cell proliferations in both systems were similar at the early stage of reprogramming (Figure 2l). These findings suggest that the enhanced activities of OCT4 and SOX2 facilitated cell cycle remodeling during reprogramming.

### 2.4 Remodeled Cell Cycle Promotes Reprogramming

**Figure 3:**
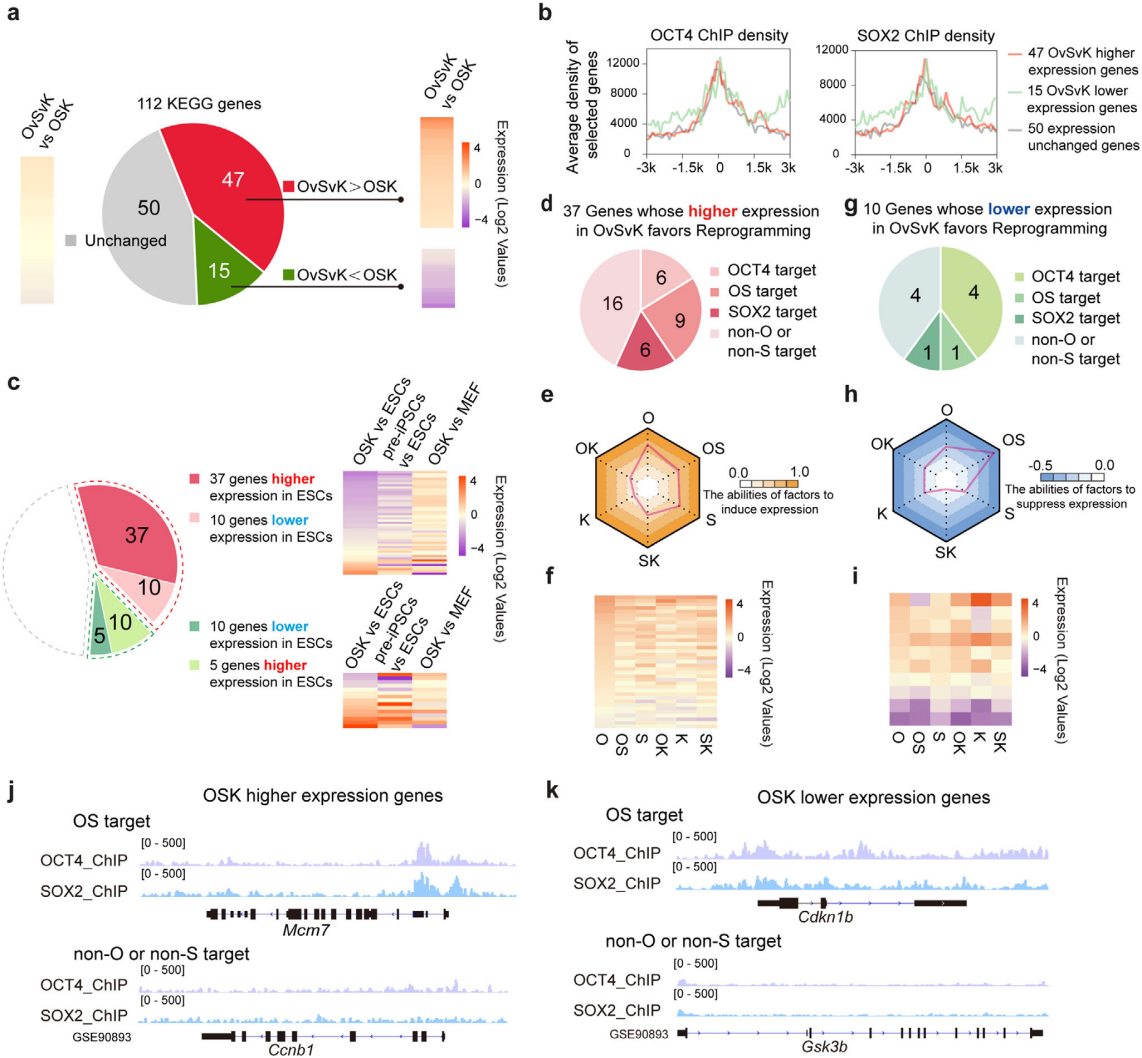
Cell cycle‐related genes connected OvSvK. a) 112 genes in the cell cycle pathway (KEGG: mmu04110) were expressed in different level between OSK and OvSvK. 47 genes showed higher expression in OvSvK, 15 genes showed lower expression, and 50 genes showed no significant difference. b) ChIP‐seq data obtained from the GEO dataset GSE90893 showed that OCT4 and SOX2 had no significant difference in binding to these three types of genes. c,d,g) Thirty‐seven of 47 genes with higher expression and 10 out of 15 genes with lower expression favored reprogramming when considering their expression in OSK day 2 and ESCs. The binding of OCT4 and SOX2 to these genes is summarized in (d,g). e,f,h,i) The abilities of O, S, K, OS, OK, and SK to regulate the expression of the 37 and 10 genes mentioned in (c) were summarized. j,k) Integrative Genomics Viewer (IGV) browser snapshots compared OS target genes and non‐target genes by ChIP–seq in OSK higher expression genes j) and OSK lower expression genes k).

Given that the cell cycle of MEFs differs significantly from that of ESCs,^[^ ^24^ ^]^ we hypothesized that remodeling the cell cycle could promote reprogramming. To further investigate the relationship among the enhanced activities of OCT4 and SOX2, the cell cycle remodeling and the promoted reprogramming, we analyzed 112 genes associated with the cell cycle pathway (KEGG: mmu04110). Among these, 47 genes exhibited higher expression, 15 showed lower expression, and 50 displayed no significant difference between OvSvK and OSK on day 2 of reprogramming (**Figure** **3**a). The upregulation of pluripotency‐related genes was comparable between OSK and OvSvK on day 2, suggesting a similar status during reprogramming (Figure 2c). Additionally, cell proliferation rates were similar in both systems, and comparison on day 2 would exclude the influence of subsequent processes (Figure 2i).

Since VP16 enhances the transcriptional activity of OCT4 and SOX2, we analyzed their binding abilities to the three groups of genes in Figure 3a based on a previously reported ChIP‐seq dataset (GSE90893).^[^ ^5^ ^]^ Our analysis confirmed the binding of OCT4 and SOX2 to these gene groups, with no significant differences observed among them (Figure 3b). Next, we compared the differentially expressed genes between OvSvK and OSK, focusing on their expression differences in OSK vs. ESCs, pre‐iPSCs vs. ESCs, and OSK vs. MEF (Figure 3c). Among the 47 genes highly expressed in OvSvK, 37 genes were upregulated in ESCs compared to OSK, suggesting these genes were conducive to reprogramming. Supporting this, most of these 37 genes also exhibited lower expression levels in pre‐iPSCs compared to ESCs (Figure 3c). Conversely, the remaining 10 genes showed higher expression in OSK than in ESCs, implying that their high expression might be detrimental or unrelated to reprogramming. We further checked 37 genes and found that over half of these genes could be upregulated by OCT4, SOX2, or OCT4+SOX2 (Figure 3d–f,j). A similar analysis was performed for the 15 genes with lower expression in OvSvK compared to OSK. We found that 10 of these genes were downregulated in ESCs compared to OSK (Figure 3c), and more than half of these genes were regulated by OCT4, SOX2, or both (Figure 3g–i,k). Therefore, the enhanced activities of OCT4 and SOX2 facilitated cell cycle remodeling by modulating the expression of cell cycle‐related genes.

### 2.5 Modulation of Cell Cycle Related Gene Expression Affects Reprogramming Efficiency

**Figure 4:**
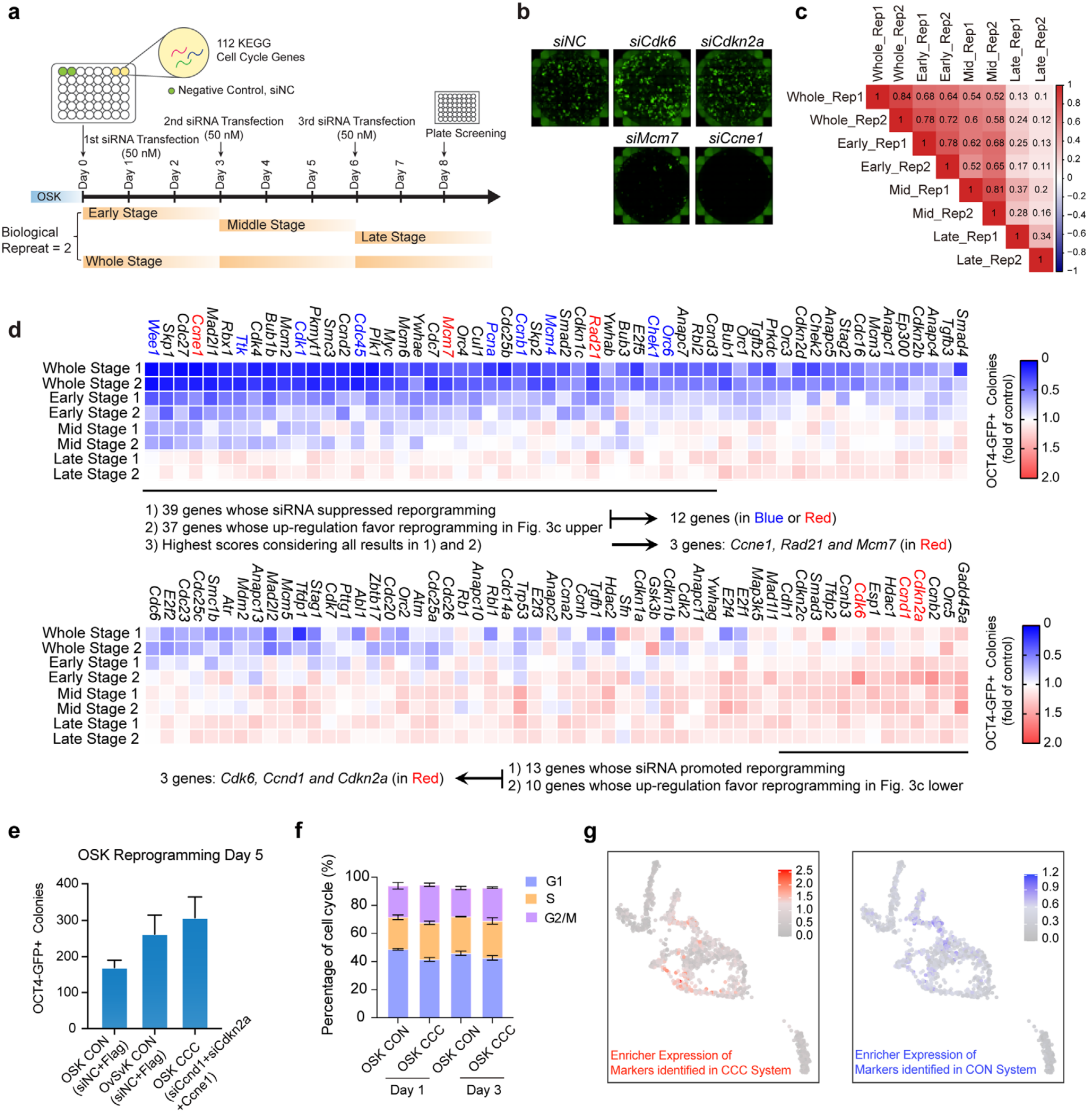
Cell cycle‐related genes accelerated reprogramming. a) Diagram illustrating the siRNA library screen. b) Representative whole well screening of OCT4‐GFP^+^ colonies in 48‐well plates (siNC, si*Cdk6*, si*Cdkn2a*, si*Mcm7*, and si*Ccne1*). c) The correlation of gene expression at different reprogramming stages. d) siRNAs were introduced three times during reprogramming in “Whole Stage” studies, while only once in the other three types of studies (Early, Mid, or Late Stage). The heatmap showed the reprogramming efficiency of eight experiments. Colony numbers were normalized to siNC. e) OCT4‐GFP+ colonies on day 5 with CON (Control, OSK with siNC and FLAG) and CCC (*Ccne1*, si*Ccnd1*, and si*Cdkn2a*) treatment. f) FACS analysis evaluated the effect of CCC treatment and the CON treatment on the cell cycle distribution of OSK cells. g) The expression of marker genes day 2 during CON treatment and CCC treatment reprogramming were presented into Figure 1g. Cell cycle modulation moved reprogramming from OSK to OvSvK.

We employed a library of three siRNAs targeting each gene in the cell cycle pathway (KEGG: mmu04110, 112 genes) for reprogramming to assess their effects on iPSCs generation (**Figure** **4**a–c). During the siRNA library screening, we categorized the genes of interest into two classes (Figure 4d). The first class of genes were whose knockdown by siRNA inhibited reprogramming and whose expression was upregulated during reprogramming. We identified 39 such genes, among which 12 exhibited higher expression in OvSvK, suggesting a beneficial role in reprogramming (Figure 4d). *Ccne1*, *Rad21*, and *Mcm7* which had the highest scores in the current analyses were selected for further studies (Figure 4d). The second class of genes were whose knockdown by siRNA, promoted reprogramming and whose expression was downregulated during reprogramming. We identified *Cdk6*, *Ccnd1*, and *Cdkn2a* among the 13 genes whose siRNAs facilitated reprogramming (Figure 4d).

For the first class of genes, we employed an overexpression approach to determine whether they could enhance reprogramming efficiency (Figure S5c, Supporting Information). Like OvSvK, we hypothesized that the overexpression of these genes in the first class would shorten the G1 phase or extend the proportion of the S phase. Conversely, for the second class of genes, we expected siRNA‐mediated knockdown to have a similar impact on the cell cycle. To validate these effects, we examined cell cycle progression following siRNA‐mediated knockdown (Figure S5a, Supporting Information) and gene overexpression (Figure S5b, Supporting Information). Although si*Cdk6* promoted reprogramming, it unexpectedly prolonged the G1 phase. Similarly, si*Rad21* also extended the G1 phase. Notably, overexpression of RAD21 had no effect on the cell cycle. Since that si*Cdk6* or si*Rad21* did not shorten the G1 phase, neither *Cdk6* nor *Rad21* was included in the final selection.

Taking into account the impact of cell cycle‐related genes on OSK reprogramming and their effects on cell cycle progression, we identified a combination of *siCcnd1*, *siCdkn2a*, and over‐expression of *Ccne1* (CCC) as an effective strategy to mimic cell cycle remodeling and facilitate reprogramming (Figure 4e,f). scRNA‐seq and qPCR results showed that *Ccne1* was upregulated during OvSvK reprogramming, with a higher expression level than in OSK. In contrast, *Ccnd1* and *Cdkn2a* exhibited a downregulation trend and were expressed at lower levels compared to OSK (Figure S5d,e, Supporting Information). These results are consistent with the promotion of OSK reprogramming by the CCC combination. MEFs with a fluorescent ubiquitination‐based cell‐cycle indicator (FUCCI) was used to indicate the cell cycle stage^[^ ^25^ ^]^ (Figure 4f). Transcriptome analysis of cells on day 2 of reprogramming, comparing those treated with control siRNAs and vectors (CON) versus those treated with *siCcnd1*, *siCdkn2a*, and *Ccne1* (CCC), suggested that the CCC shifted reprogramming from the OSK route to the OvSvK route (Figure 4g). These findings demonstrate that cell cycle remodeling links the enhanced activities of OCT4 and SOX2 with promoted reprogramming.

### 2.6 OvSvK Promotes Reprogramming by Opening Chromatin on Direct Targets

**Figure 5:**
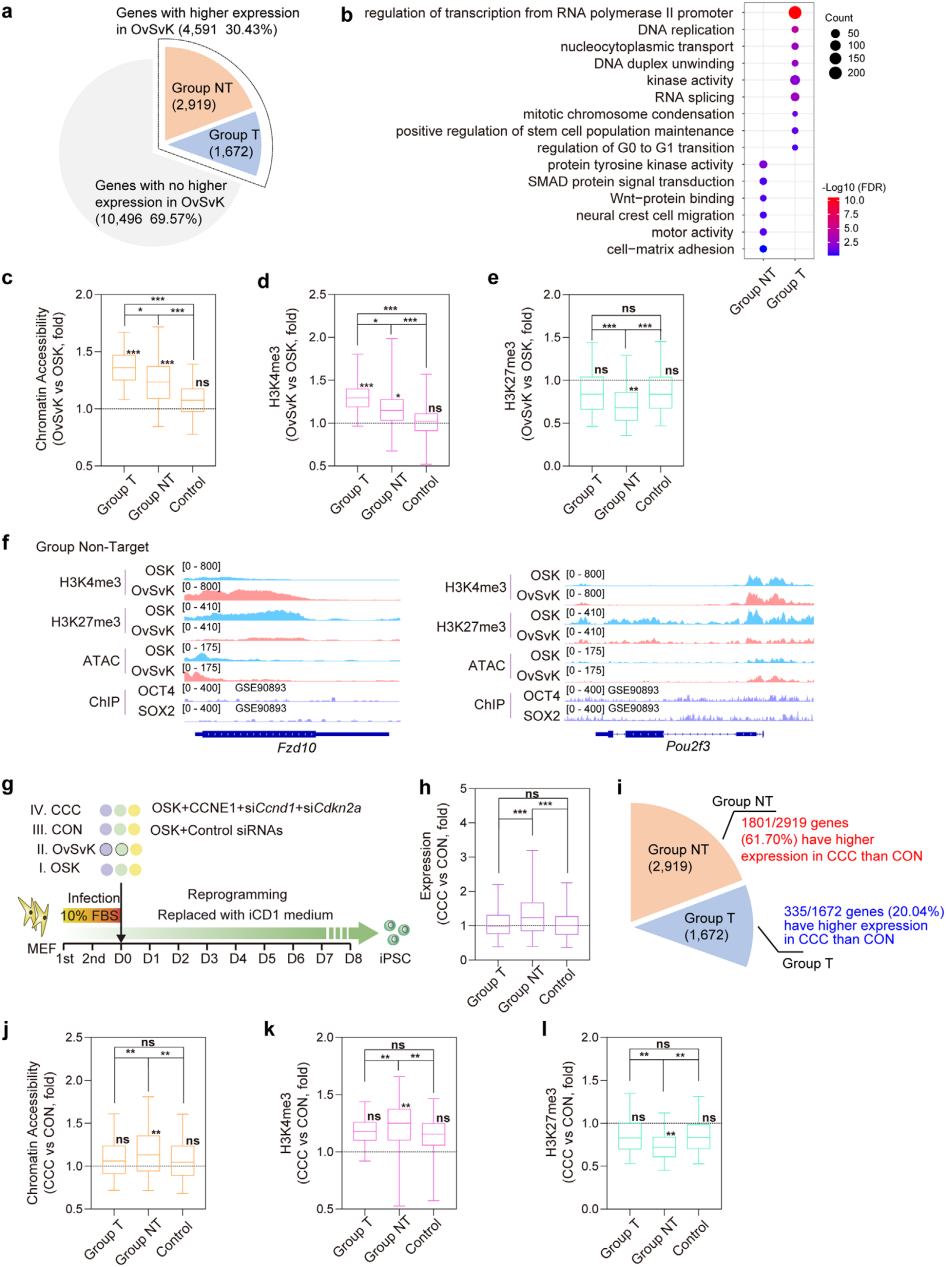
Transcriptional activation and chromatin opening contributed to the promotion of reprogramming. a) Two criteria were used to classify all identified genes into three groups. Two criteria are 1) Higher expression on day 2 during OvSvK reprogramming than OSK (Group T and Group NT) and 2) Maximum activation on day 2 during reprogramming with O, S, OS, or OSK (Group T). b) GO analysis of the genes in Group T and Group NT. c–e) Chromatin accessibility (c) and H3K4me3 (d) and H3K27me3 (e) modification of the three groups of genes were compared during OSK and OvSvK reprogramming. The * on the top bars indicated the comparisons between groups and “0”; the * on the top lines indicated the comparisons between different groups. f) Integrative Genomics Viewer (IGV) tracks showed the enriched regions of Fzd10 and Pou2f3 in Group NT. g) Schematic diagram of CCC and CON reprogramming systems. h,i) The expression of genes was compared in CCC and CON systems. The genes in Group NT had higher expression in CCC systems at h) higher intensity and i) at higher frequency. j–l) Chromatin accessibility (j), H3K4me3 (k), and H3K27me3 (l) of genes were compared in CCC and CON systems. The genes in Group NT had higher H3K4me3 and lower H3K27me3. The * on the top bars indicated the comparisons between groups and “0”; the * on the top lines indicated the comparisons between different groups.

To investigate the mechanisms underlying the rapid reprogramming by OvSvK, we first considered the factor that VP16 enhances the activities of OCT4 and SOX2. We identified 4,591 genes that exhibited higher expression on day 2 in OvSvK compared to OSK (**Figure** **5**a; Figure S6a, Supporting Information). Based on ChIP‐seq data, these genes were classified into two groups: targets of OCT4 or SOX2 (Group T, 2,919 genes) and non‐targets (Group NT, 1,672 genes) (Figure 5a; Figure S6b–e, Supporting Information). The remaining 10,496 genes, with similar or lower expression in OvSvK, were designated as Control genes in subsequent analyses. The Group NT genes were not upregulated during OSK reprogramming (Figure S6f, Supporting Information) and exhibited moderate upregulation by OCT4, SOX2, or their combined activity (Figure S6g, Supporting Information).

GO analysis indicated that Group T genes were enriched for DNA replication, RNA polymerase II, and cell cycle‐related functions (Figure 5b), consistent with the enhanced activities of OCT4 and SOX2 in remodeling the cell cycle (Figure 3). In contrast, Group NT genes were enriched for multicellular organism development and extracellular matrix, suggesting their potential roles in regulating MET and EMT during reprogramming (Figure 5b).

We performed ATAC‐seq and CUT&Tag to investigate the differences in epigenetic modifications between OSK and OvSvK reprogramming. We observed higher chromatin accessibility, increased levels of H3K4me3, and decreased levels of H3K27me3 during reprogramming with OvSvK (Figure S7a–c, Supporting Information). However, we observed no significant difference in H3K9me3 and H3K36me3 (Figure S7d,e, Supporting Information), which is reasonable since barriers associated with these two epigenetic modifications can be efficiently overcome by vitamin C, a key component of the current reprogramming medium.^[^ ^26^, ^27^ ^]^ We also observed no significant difference in H3K27ac levels between OSK and OvSvK systems (Figure S7f, Supporting Information). We further analyzed the expression changes of imprinted genes along the reprogramming trajectory in the OSK and OvSvK systems using single‐cell sequencing data, and found that the expression trends of imprinted genes were highly similar between the two systems (Figure S8a, Supporting Information). We further analyzed the expression dynamics of imprinted genes along the reprogramming trajectories of the OSK and OvSvK systems using single‐cell sequencing data. Most of these genes exhibited similar expression trends between the two systems (Figure S8a, Supporting Information). Specifically, 67 imprinted genes showed positive correlations, with 63 of them reaching statistical significance (*p* < 0.05). Additionally, dynamic time warping (DTW) analysis was conducted on 58 imprinted genes with substantial expression levels.^[^ ^28^ ^]^ This analysis demonstrated a high degree of similarity in expression trends, as reflected by lower DTW distances for imprinted genes compared to the overall gene set (Figure S8b, Supporting Information). Among the 58 analyzed genes, 42 genes including *Igf2* and *Peg13* had DTW distances below 250, while the remaining 16 genes, such as *Cdkn1c* and *Ddc*, had distances above 250 (Figure S8c–e, Supporting Information). In addition, we assessed the methylation status of imprinted genes like *Mest* and *Snrpn* in iPSC lines derived from both OSK and OvSvK reprogramming systems using bisulfite sequencing. The results showed no notable differences in methylation patterns between the two systems (Figure S8f, Supporting Information).

Further analysis indicated that the chromatin accessibility of genes in Group T and Group NT was higher than that observed during OSK reprogramming (Figure 5c), consistent with the global chromatin opening observed in Figure S7a (Supporting Information). Additionally, the levels of chromatin opening of genes in Group T were larger than those in Group NT (Figure 5c; Figure S7g, Supporting Information). Similar differences were observed with H3K4me3. Genes in Group T and Group NT, but not the Control group, exhibited higher levels of H3K4me3 in OvSvK compared to those in OSK, with a greater elevation in Group T than in Group NT (Figure 5d; Figure S7h, Supporting Information). Lower levels of H3K27me3 in OvSvK were observed only in genes in Group NT (Figure 5e; Figure S7i, Supporting Information). Representative genes *Fzd10* and *Pou2f3* in Group NT were selected to illustrate these epigenetic differences (Figure 5f). Thus, enhanced activities of OCT4 and SOX2 promote reprogramming by activating direct targets in Group T, which correlates with increased chromatin accessibility and elevated H3K4me3 levels. Meanwhile, the upregulation of genes in Group NT is not a direct result of enhanced activities of OCT4 and SOX2, but is connected with the reduced levels of H3K27me3.

Previous studies have shown that transcription factors such as OCT4 and SOX2 not only bind to gene transcription regions to activate their targets but also bind to enhancer regions to enhance gene transcription.^[^ ^29^ ^]^ Therefore, based on enhancers associated with MEF and mouse ESCs from EnhancerAtlas 2.0,^[^ ^30^ ^]^ we analyzed the chromatin accessibility of the corresponding regions in our reprogramming system. We found that the overall difference in the accessibility of these enhancers between OvSvK and OSK was not significant (Figure S9a, Supporting Information), and the expression differences of genes corresponding to these different enhancers were also not substantial either (Figure S9b, Supporting Information).

We also analyzed the ATAC‐seq dataset from a previous study (GSE93029)^[^ ^31^ ^]^ to explore the chromatin accessibility of the targets of OCT4, SOX2, and KLF4. The analysis revealed that the chromatin accessibility for each transcription factor was higher at its target sites compared to non‐target sites (Figure S9c–e, Supporting Information). Moreover, the targets of OCT4 and SOX2 exhibited higher chromatin accessibility in OvSvK compared to OSK (Figure S9f, Supporting Information), suggesting that OCT4‐VP16 and SOX2‐VP16 have greater abilities to open chromatin than their unmodified counterparts. The fact that KLF4 showed a moderate increase in OSK was consistent with their unmodified counterparts of OvSvK to induce reprogramming (Figure S9f, Supporting Information). To further compare the differences in downstream gene regulation mediated by O/Ov and S/Sv in OSK and OvSvK, we performed CUT&Tag analysis for OCT4 and SOX2 in both reprogramming conditions. More than 50.64% peaks in O and Ov datasets were identified in each other, and ≈56.94% peaks in S and Sv datasets were identified in each other (Figure S10a, Supporting Information). Signal analysis of OCT4 and SOX2 peaks revealed that the average signal intensity in OvSvK was higher than that in OSK (Figure S10b, Supporting Information). Furthermore, an analysis of chromatin accessibility at OCT4 and SOX2 peak regions indicated that OvSvK exhibited a higher average signal intensity compared to OSK (Figure S10c, Supporting Information). We also compared the regulatory effects of OCT4, OCT4‐VP16, SOX2, and SOX2‐VP16 on three groups of genes presented in Figure 3a. We observed 50 genes with unchanged expression, 47 genes upregulated in OvSvK, and 15 genes downregulated in OvSvK. The results showed that VP16 fusion did not significantly alter OCT4 binding across the three gene groups. In contrast, SOX2‐VP16 exhibited increased binding intensity for both upregulated and downregulated genes (Figure S10d,e, Supporting Information). In summary, the abilities of these factors to promote chromatin opening, along with their enhanced capabilities through VP16 fusion, which partially explained the upregulation of genes in Group T and the increased chromatin accessibility in OvSvK.

### 2.7 OvSvK Activates Indirect Targets by Remodeling Cell Cycle

We then aimed to distinguish the beneficial roles played by the enhanced activities of OCT4 and SOX2 from those of the remodeled cell cycle during reprogramming. To validate the relationship between the upregulation of genes in Group T and Group NT and changes in the cell cycle, we compared the expression of these genes during reprogramming with the control (CON) or the new combination (CCC) (Figure 5g). The average expression intensity of the upregulated genes in Group NT genes in the CCC system was greater than that of Group T genes (Figure 5h). In fact, only 335 out of 1672 genes (20.04%) in Group T had higher expression in CCC system, while 1801 of 2919 genes (61.70%) in Group NT had higher expression (Figure 5i).

Chromatin accessibility and histone methylation were also assessed on day 2 during CCC and CON reprogramming (Figure 5j–l). The epigenetic remodeling between OvSvK and OSK was compared with that between CCC and CON. Unlike the comparison between OvSvK and OSK, the intensity of chromatin opening in Group T genes in the CCC system was relatively low and similar to that in the control group (Figure 5j; Figure S7j, Supporting Information). Similar observations were made for histone methylation (Figure 5k,l; Figure S7k,l, Supporting Information). In summary, the epigenetic remodeling of genes in Group T was only identified in OvSvK, not in the CCC system.

On the other hand, the differences in chromatin accessibility and histone methylation of genes in Group NT were consistent. Genes in Group NT exhibited greater chromatin accessibility, higher H3K4me3 levels, and lower H3K27me3 levels in CCC system (Figure 5j–l; Figure S7j–l, Supporting Information), similar to the differences observed in OvSvK compared to OSK (Figure 5c–e; Figure S7g–i, Supporting Information). These results indicated that the remodeled cell cycle could affect the epigenetic modification of Group NT genes rather than that of Group T, thereby influencing gene expression and enhancing reprogramming efficiency.

### 2.8 A Shortened G1 Phase Affects the Restoration of Histone H3K27me3

**Figure 6:**
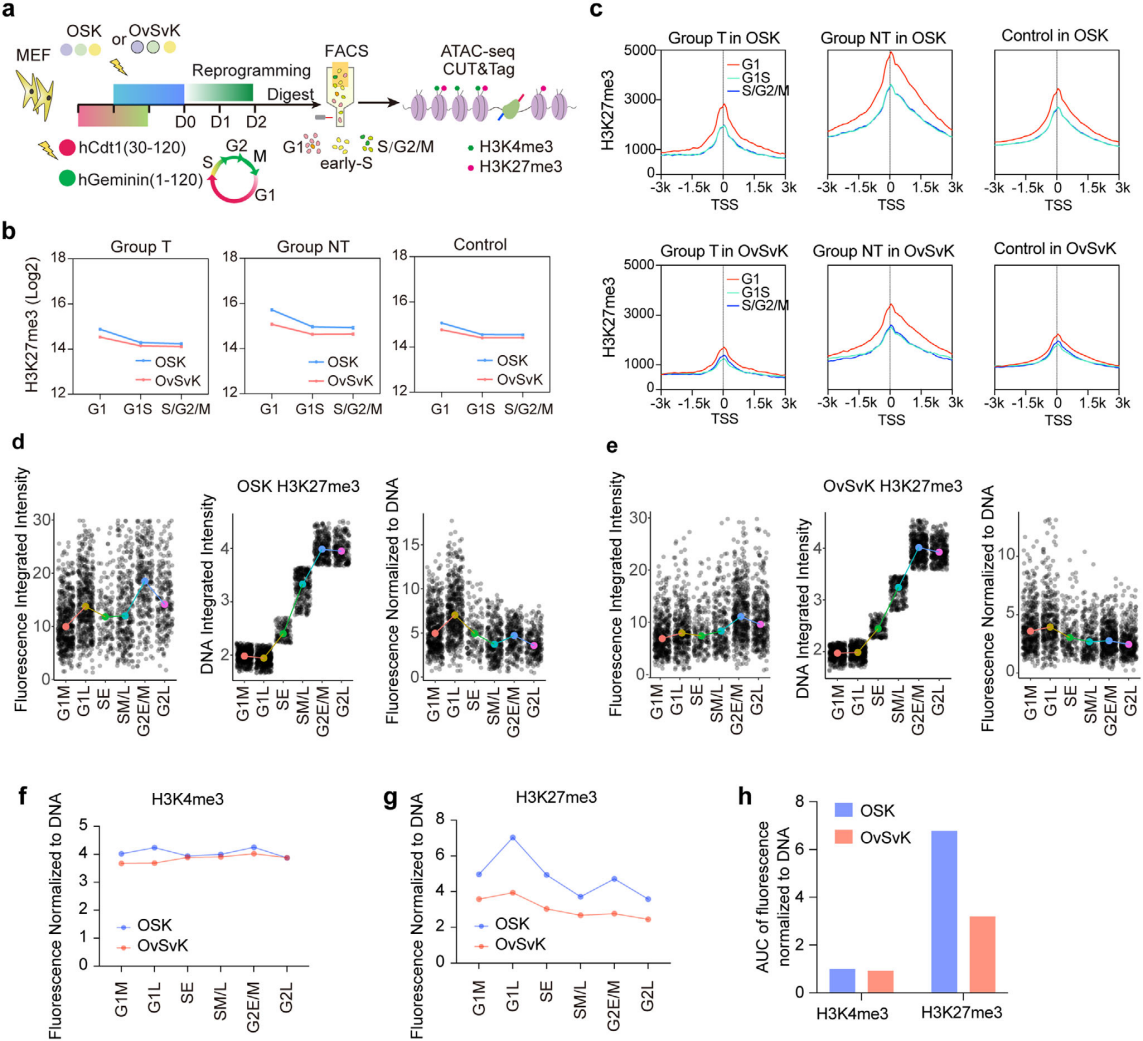
The shortening of the G1 phase affected the recovery of H3K27me3 and promoted reprogramming. a) Schematic diagram of ATAC‐seq and CUT&Tag in different cell cycle stages of OvSvK and OSK reprogramming. b,c) Difference of total H3K27me3 (b) and H3K27me3‐enriched signals around TSS (c) for three groups of genes at different cell cycle stages during reprogramming. d,e) Statistics of integrated fluorescence intensity of H3K27me3 (d,e, left), integrated fluorescence intensity of DNA (d, e, middle), and relative fluorescence intensity of H3K27me3 normalized to DNA (d, e, right) across various cell cycle stages. f,g) The DNA‐normalized relative fluorescence intensity of H3K4me3 and H3K27me3 across the cell cycle stages. h) Area under the curve (AUC) analysis of (f) and (g).

To further explore the impact of cell cycle changes on epigenetic remodeling, we performed ATAC‐seq and CUT&Tag to investigate chromatin accessibility and histone modifications at different cell cycle stages. FUCCI‐MEFs were infected with OSK or OvSvK and collected on reprogramming day 2. Cells were sorted by fluorescence‐activated cell sorting (FACS) into different phases: G1, early S, and S‐G2‐M phases (**Figure** **6**a).

Above studies showed that changes in H3K27me3 levels of Group NT genes were more significant following cell cycle alterations (Figure 5e). Therefore, we first focused on the pattern of H3K27me3 changes throughout the cell cycle. We found that the H3K27me3 modification level in Group NT genes was higher than in Group T genes in both OSK and OvSvK (Figure 6b,c). Previous studies indicated the maintenance of H3K27me3 occurs in G1 phase,^[^ ^21^ ^]^ and we observed a decrease in H3K27me3 density in the early S phase. As the cell cycle progressed from the S‐G2‐M phases into the G1 phase, H3K27me3 modification increased in both OSK and OvSvK systems, particularly in Group NT genes (Figure 6b,c). This increase was more significant in OSK compared to OvSvK (Figure 6b,c). We also analyzed chromatin accessibility and H3K4me3 modifications throughout the cell cycle. The chromatin accessibility and H3K4me3 modification of genes in Group T were almost constant in G1, early‐S, and S‐G2‐M phase, regardless of whether the OSK or OvSvK system was analyzed. Therefore, chromatin accessibility and H3K4me3 modification were stable during cell proliferation, which was not affected by the cell cycle modulation. Similar observation was obtained with genes in Group T and Group Control (Figure S11a–d, Supporting Information).

The increase in H3K27me3 in the G1 phase was more significant in OSK compared to OvSvK (Figure 6b,c), which is consistent with the decrease in H3K27me3 in the OvSvK system (Figure 5e,i). Since epigenetic modifications, such as DNA methylation and histone methylation, might be incomplete during the S phase and require further supplementation during G2/M phase or even the new G1 phase,^[^ ^18^, ^19^, ^20^, ^21^ ^]^ we proposed that the shortened G1 phase in the OvSvK system impaired the full recovery of epigenetic modifications during cell proliferation. For example, the larger increase in H3K27me3 in the G1 phase in the OSK system suggests incomplete inheritance during the S phase and a further supplementation requirement in the OvSvK system. The shortened G1 phase impaired further supplementation, leading to a reduced difference between G1 and other phases. This incomplete inheritance subsequently led to lower H3K27me3 levels of Group NT genes in OvSvK system.

To confirm the hypothesis above, we developed a method for identifying cell cycle stages using image recognition. This method utilizes parameters such as the fluorescence intensity of DAPI and cell morphology to display cells in 2D images according to their cell cycle stages (Figure S12a,b, Supporting Information). We performed immunofluorescence staining for H3K27me3 and H3K4me3 in OSK and OvSvK systems and mapped the cells to their corresponding cell cycle stages (Figure 6d,e; Figure S12c–h, Supporting Information). Given the limited cell number in the M and early‐G1 phases, we focused our analysis on cells in the middle‐G1 (G1M), late‐G1 (G1L), early‐S (SE), middle/late‐S (SM/L), early/middle‐G2 (G2E/M), and late‐G2 phases (G2L).

Both H3K27me3 levels and DNA content gradually increase from G1 to G2 phase. After normalizing the H3K27me3 levels to DNA content, we found that H3K27me3 peaked during the late‐G1 phase and began to decline upon entering the S phase (Figure 6d,e). These results suggested that the restoration rate of H3K27me3 is slower than the rate of DNA synthesis, leading to the loss of H3K27me3 modifications at the end of the S phase. Such incomplete inheritance required G2 and additional G1 phases to complete, which might be impaired when G1 phase was shortened. We also compared the changes in H3K27me3 relative to DNA content between OSK and OvSvK and found that the changes in OvSvK were more gradual (Figure 6g,h), which was consistent with the smaller elevation of H3K27me3 in the G1 phase (Figure 6b).

We also analyzed H3K4me3 throughout the cell cycle with a similar image‐based method and found that H3K4me3 exhibited much smaller changes compared to H3K27me3 (Figure 6f,h; Figure S12e–h, Supporting Information). In addition, there were no significant differences between OSK and OvSvK systems. These results suggested that the inheritance of H3K4me3 is quicker than that of H3K27me3. The G1 phase was not required for the restoration of H3K4me3, and the shortened G1 phase in OvSvK system should not affect H3K4me3.

Furthermore, we performed similar cell cycle image‐based analyses for the CCC and CON (Figure S13a–h, Supporting Information). We observed that, compared to CON, CCC displayed a more gradual change in H3K27me3 levels, whereas H3K4me3 levels remained unchanged (Figure S13i–k, Supporting Information). Together, these observations suggested cell cycle remodeling affects the stability of histone modification inheritance. The shortened G1 phase perturbed the balance of H3K27me3, thereby promoting reprogramming.

## 3 Discussion

**Figure 7:**
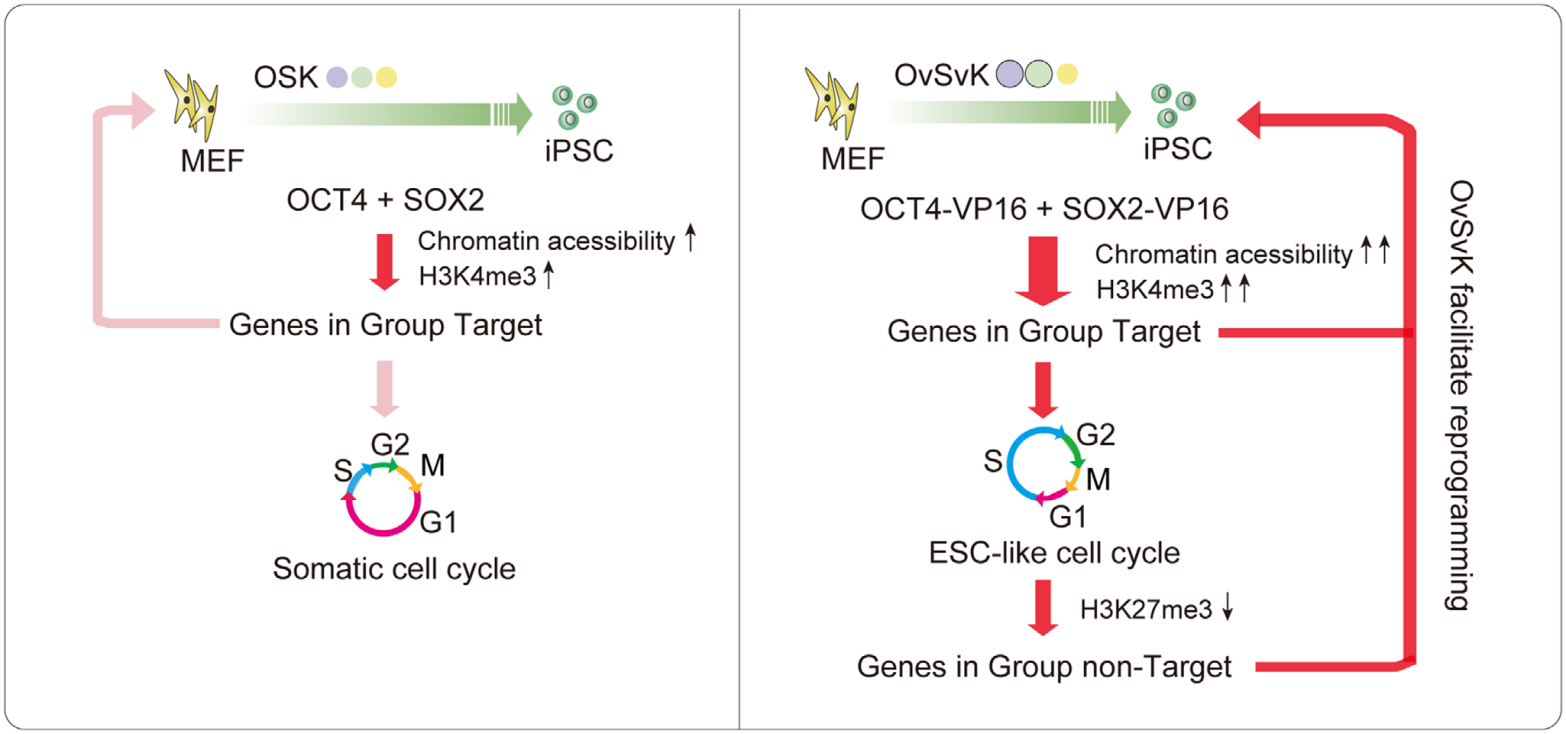
A summary of OvSvK reprogramming. In traditional OSK reprogramming, MEF cells display a somatic cell cycle characterized by a long G1 phase. OCT4 and SOX2 facilitate opening chromatin and increase H3K4me3 level at their direct targets (left panel). During the enhanced OvSvK reprogramming, MEF cells exhibit an ESC‐like cell cycle with a shortened G1 phase and an extended S phase. Fusion with VP16 enhances OS transcriptional ability, leading to higher chromatin accessibility and H3K4me3 levels. The remodeled cell cycle in OvSvK also disrupts histone inherence, resulting in decreased H3K27me3 levels in Group NT genes (right panel).

In this study, we established a new reprogramming system, OvSvK, which derived germline‐competent iPSCs within 4 days and used it to investigate new mechanisms for the establishment of pluripotency. We found that VP16 enhanced the gene expression of the direct targets of OCT4 and SOX2, including cell cycle‐regulated genes, which led to a shortened G1 phase. Further studies showed the remodeling of cell cycle led to impaired restoration of H3K27me3 modification during cell proliferation, causing the up‐regulation of genes in Group NT (**Figure** **7**).

It is known that the balance among key transcription factors is essential for the cell fate decisions and conversions. During the establishment of pluripotency, OCT4 and SOX2 have been suggested to function as pioneer factors.^[^ ^4^, ^32^ ^]^ KLF4 also plays a crucial role in reprogramming, and our earlier studies confirmed that KLF4 promotes the mesenchymal‐to‐epithelial transition (MET).^[^ ^33^ ^]^ Interestingly, we found that altering the order of transcription factor expression can lead to differences in reprogramming efficiency, even when using the same combination of factors.^[^ ^7^ ^]^ These findings suggest that although OSK is considered a core set of pioneer reprogramming factors, their functional contributions vary depending on the specific reprogramming system and factor combinations. In our previous single‐cell sequencing study on OSK‐mediated reprogramming,^[^ ^6^ ^]^ we observed that KLF4 induced a distinct non‐reprogramming (NR) branch characterized by *Cd34* ^+^/*Fxyd5* ^+^/*Psca* ^+^ cells. A similar NR branch was identified in this study. However, in prior research, when we attempted to regulate KLF4 expression levels, we found a positive correlation between the generation of GFP^+^ cell and the emergence of CD34^+^ NR cells.^[^ ^6^ ^]^ This suggests that KLF4 plays a dual role in reprogramming. Other studies have shown that VP16 domain enables OCT4 and SOX2 to be much stronger transcriptional activators during reprogramming. In this study, we demonstrated these enhanced activities of OCT4 and SOX2, but not KLF4, efficiently promoted reprogramming.

In addition to the use of VP16, transcription factor engineering methods have been employed in numerous studies to promote cell fate conversion. Mutagenesis and screening of OCT4 and SOX2 protein structures have led to the development of engineered variants such as ePOU and eSOX, which enhance somatic cell reprogramming efficiency.^[^ ^34^, ^35^ ^]^ Additionally, studies on the SOX family of proteins have shown that mutating several sites on the SOX17 protein leads to the generation of the SOX17FNV variant, which exhibits a stronger capacity for establishing pluripotency compared to SOX2.^[^ ^36^ ^]^ The intrinsically disordered regions (IDRs) within the protein sequence are involved in liquid‐liquid phase separation (LLPS). OCT4 phase separation can form condensates with the mediator coactivator and regulate topologically associating domain (TAD) reorganization.^[^ ^37^, ^38^ ^]^ Recent studies have found that increasing the aromatic dispersion of multiple human TFs enhances their transcriptional activity and promotes LLPS in vitro.^[^ ^39^ ^]^ These engineering of transcription factor amino acids has facilitated their role in cell fate conversion.^[^ ^39^ ^]^ In the future, we could further investigate other methods of modifying reprogramming factors to assess their effects on pluripotency establishment, particularly in relation to the cell cycle and epigenetic modifications.

Although OvSvK and OSK systems share the same NR route, their reprogramming routes are different. By using different methods to compare the transcriptomes in these two systems, we identified the remodeling of cell cycle and facilitated the shortening of G1 phase in OvSvK. We attribute the accelerated reprogramming in OvSvK to two types of effects: the direct upregulation of downstream targets of OCT4 and SOX2, and indirect effects mediated by the remodeling of the cell cycle.

Accelerated cell proliferation facilitates cell fate conversion processes including reprogramming, differentiation, and transdifferentiation,^[^ ^24^, ^40^ ^]^ further supporting the connection between the remodeled cell cycle and epigenetic modification. It is reasonable to connect changes in cell cycle dynamics and epigenetic remodeling through the inheritance of epigenetic modifications during cell proliferation.

The incomplete inheritance of DNA methylation requires further supplementation in the G1 phase.^[^ ^18^, ^19^ ^]^ Thus, shortening the G1 phase should impair the restoration of DNA methylation to basal levels, subsequently inducing DNA demethylation during cell proliferation. In fact, by using shRNA to knockdown *P53* expression, we were able to shorten the G1 phase and induce passive DNA demethylation in MEFs.^[^ ^18^ ^]^ The inheritance of histone methylation is also complex and not fully elucidated.^[^ ^20^, ^21^ ^]^ However, whether shortening the G1 phase could affect epigenetic modifications depends on their inheritance rates during the S phase. If the epigenetic modifications, such as H3K4me3, restore quickly during the S phase, their levels remain constant throughout cell cycle when normalized to DNA content. If the epigenetic modifications, like H3K27me3, restore slowly during the S phase, their levels experience wide oscillations throughout the cell cycle, with a significant elevation expected during the G1 phase. Additionally, shortening the G1 phase does not enhance such oscillations, because the elevation in G1 phase is reduced, and the related epigenetic modification becomes easier to inherit during S phase. This explains the narrow oscillation observed in OvSvK and CCC systems.

Reduced levels of H3K27me3 were induced by the shortened G1 phase in the OvSvK and CCC systems. However, this decrease was preferentially observed in genes in Group NT rather than in genes in Group T. The underlying mechanisms require further investigation to elucidate. Currently, two possibilities have been considered. First, the genes in Group NT bear higher levels of H3K27me3 than those in other two groups (Figure S7g, Supporting Information). Higher levels of H3K27me3 are more difficult to inherit during S phase, which results in significant demethylation of these genes. Second, the genes in Group NT have a large overlap with the genes more likely to undergo DNA demethylation, both in active DNA demethylation induced by TET1 and passive DNA demethylation induced by shRNAs against *Dnmt1* and *P53* (data not shown),^[^ ^41^ ^]^ suggesting a correlation between the inheritance of DNA methylation and H3K27me3.

In the current study, the upregulation of genes in Group T was attributed to the binding of OCT4 and SOX2 followed by transcriptional activation. Furthermore, the chromatin accessibility of these genes increased during OvSvK reprogramming, confirming the pioneering functions of OCT4 and SOX2. Moreover, OCT4 and SOX2 serve as bookmarking factors during cell proliferation.^[^ ^42^, ^43^, ^44^ ^]^ Thus, the interaction between OCT4/SOX2 and chromatin after the S phase and during M phase may affect the reestablishment of chromatin structure, contributing to the inheritance of histone methylation as well as DNA methylation.

In summary, by modulating the balance among the transcription factors, reprogramming was optimized by remodeling of the cell cycle and epigenetic modifications. The connection between these two types of remodeling may provide new insights into the inheritance of epigenetic modifications during cell fate transitions.

## 4 Experimental Section

### Cell Lines and Primary Cultures

Plat‐E cell line was a gift from The Fourth Military Medical University. Primary MEFs were derived from 13.5 d.p.c. Plat‐E and MEF cells were maintained in high glucose DMEM supplemented with 10% fetal bovine serum, GlutaMAX and NEAA. All cell types tested negative for mycoplasma, according to the results of the mycoplasma detection kits (Lonza).

### Animals

All of the mouse work performed in this study received approval from the Animal Ethics Committee in Guangzhou Institutes of Biomedicine and Health, Chinese Academy of Sciences (IACUC 2007007), to ensure compliance with ethical principles and regulations. The OG2 transgenic mice (CBA/CaJ × C57BL/6J) used in this study originated from the Jackson Laboratory (Mouse strain datasheet: 004654), while CD‐1 (Stock No. 201) and 129 (Stock No. 217) mice were obtained from Beijing Vital River Laboratory Animal Technology Co., Ltd. The mice were housed in a temperature‐controlled room maintained at 22–23 °C and exposed to a 12‐h light/dark cycle between 07:00 and 19:00, with free access to food and water.

### Generation of iPSCs

Plat‐E cells were seeded at density of 8 × 10^6^ cells per 100 mm dish and pMXs‐based retroviral vectors were introduced into plat‐E using PEI (Polyethylenimine, Polysciences, Cat# 23966) on the next day. 10–12 h after transfection, supernatants were discarded and replaced by fresh medium. Viral supernatants were collected at 24 and 48 h after adding fresh medium. OG2 MEFs were plated at 4000–5000 cells/cm^2^ the day before infection. Fresh viral supernatants were mixed with fresh medium and infect MEFs supplemented with 4 µg ml^−1^ polybrene. MEFs were infected twice with equal volumes of virus‐containing supernatants, once every 24 h, to ensure consistent infection conditions across experimental groups. At the end of the twice infection, viral supernatants were removed and iCD1 medium was added for reprogramming. This day defined as day 0 post‐infection. iCD1 medium was changed every day, until the GFP^+^ clone showed up.

To identify the pluripotency of OvSvK derived iPSCs, chimera experiments were performed. Because exo‐retrovirus silence could indicate iPSCs generation, OG2 MEF were infected with OvSvK and pMXs‐DsRed. Reprogramming cells at day 4, day 5 or day 6 were digested by 0.25% trypsin and *Oct4*‐GFP positive/DsRed negative cells were sorted by flow cytometry. After sorting, iPSCs were put on ice ready for blastocyst injection.

### Single‐cell RNA‐seq

OvSvK reprogramming cells were digested by 0.25% trypsin and washed twice with DPBS. Single cells were captured using the Fluidigm C1 Single‐Cell Auto Prep System. 10–17 µm C1 chips for reprogramming cells at day 0, day 1, day 2, and day 3. At day 4, reprogramming cells were captured both 5–10 µm and 10–17 µm C1 chips. After cell capture, chips were observed under the microscope to exclude capturing multiple cells. Cell lysis and cDNA synthesis were performed on‐chip and following the manufacturer's instructions. After cDNA harvesting, the concentrations were analysis with PicoGreen (Thermo Fisher) and 0.5 ng amplified products were used for Nextera XT library preparation and following the manufacturer's instructions. The single‐cell libraries were quantified by Quant‐iT dsDNA Assay Kit, high sensitivity (Thermo Fisher Scientific) on Qubit 2.0 (Thermo Fisher Scientific), and then sequenced on illumina NextSeq 500.

Raw bcl files were converted to fastq files by bcl2fastq (v1.84, illumina). Sequencing data were checked by FastQC and aligned to mouse reference genome mm10 (GRCm38.p5). Reads were annotated with ensemble database (Ensemble release 86) by RSEM.^[^ ^45^ ^]^

### Cell Cycle Analysis with Propidium Iodide (PI)

Reprogramming cells were gently trypsinized, and cells pellets were collected every 1 × 10^6^. Resuspended the pellets with 250 µL PBS and added 750 µL cold alcohol to fixed cell in final 75% alcohol at 4 °C overnight. On the next day, fixed cells were washed twice in cold PBS and then using 50–100 µg ml^−1^ RNaseA digested for 30 min in 37 °C water bath before staining. Cells were stained using 50–100 µL g ml^−1^ PI for 30 min, and ready to analyze on flow cytometry.

### siRNA Library Screen

The siRNA library contained 112 cell cycle‐related genes that were chosen based on the KEGG cell cycle pathway (KEGG: mmu04110). The siRNA library was synthesized by RIBOBIO, and each gene contained three different siRNAs. For each gene, these three siRNAs were mixed 1:1:1 at a final concentration of 20 µm. MEFs were plated in 48‐well plates at a density of 5 × 10^3^ cells per well. On the next day, MEFs were infected with OSK virus twice and once every 24 h. On day 0, cells were transfected with siRNA using Lipofectamine RNAiMAX (Thermo Fisher Scientific; Cat# 13778150). Briefly, 225 µL of iCD1 medium was added to each well of a 48‐well plate before transfection, and a 25 µL mixture was added to each well to obtain a final siRNA concentration of 50 nm. Transfected cells were changed to fresh iCD1 medium after 24 h. For multiple transfections, the operation was repeated at the corresponding time. At the end of reprogramming, we screened whole plates using a Nikon living‐cell station. iPS colonies were analyzed with ImageJ. Briefly, the original png format pictures were opened and converted to 8‐bit pictures. Thresholds (60/255) were selected, and then, Analyze Particles was selected using Size (550‐infinity). Counting colonies of several wells under a microscope was also necessary to ensure the accuracy of image analysis.

### ATAC‐Seq of Cell Cycle Phase‐Specific Cells

MEFs were infected with OSK or OvSvK, and also infected with both hCdt1‐mCherry and hGeminin‐mCitrine which is called the FUCCI. After infection, iCD1 medium was changed on day 0. On day 2, reprogrammed cells overexpressing FUCCI were trypsinized and sorted using flow cytometry. Three groups were as follows: mCherry positivity indicated G1 phase, mCherry and mCitrine double positivity indicated early‐S phase, and mCirtine positivity indicated S‐G2‐M phase. After sorting, the cells were washed once with DPBS. A total of 5000 cells of each of the three groups and unsorted control cells were collected by centrifugation. Cell pellets were lysed by resuspension in 50 µL of cold lysis buffer (10 mM Tris·Cl pH 7.4, 10 mM NaCl, 3 mM MgCl2, 0.1% (v/v) Igepal CA‐630). The cells were lysed on ice for 10 min, and then, nuclei were collected by centrifugation. The supernatant was discarded, and nuclei were kept on ice until the next step. ATAC‐seq libraries were constructed by the TruePrepTM DNA Library Prep Kit V2 for Illumina (Vazyme TD501) according to the manufacturer's instructions. The library was quantified with the VAHTS Library Quantification Kit for Illumina (Vazyme) on Qubit 2.0 (Thermo Fisher Scientific) and then sequenced on an Illumina NovaSeq 6000 using 150 bp paired‐end reads.

Raw ATAC‐seq reads were trimmed and filtered for quality control using Trim Galore v0.6.7. Clean paired‐end reads were first mapped to mouse genome mm10 using Bowtie2 (v2.4.5) with parameters ‘–very‐sensitive‐X 2000’. PCR duplicates were removed with sambamba (V0.6.8). Only concordantly mapped reads were used for further analyzing. Peak calling was performed with MACS2 with parameters ‘—keep‐dup all ‐q 0.05 ‐f BEDPE’. ATAC‐seq peaks were annotated to the nearest gene using R package ChIPseeker.

### CUT&Tag

OSK and OvSvK reprogramming cells were digested in 0.25% trypsin. A total of 5 × 10^4^–8 × 10^4^ cells were prepared for one library. Cells were centrifuged, and supernatants were discarded for preparation. CUT&Tag libraries were constructed by the Hyperactive Universal CUT&Tag Assay Kit (Vazyme, TD903) following the manufacturer's instructions. A total of 0.5 µl of H3K4me3 antibody (Abcam, Cat# ab8580), 1 µl of H3K27me3 antibody (CST, Cat# 9733S), 0.5 µl of H3K9me3 antibody (Abcam, Cat# ab8898), 0.5 µl of H3K36me3 antibody (Abcam, Cat# ab9050), 0.5 µl of H3K27ac antibody (Abcam, Cat# ab4729) or 1 µl of Flag antibody (CST, Cat# 14793), were used for one sample. The library was quantified using Qubit 2.0 (Thermo Fisher Scientific) and Qsep1 (Bioptic, Inc.) and then sequenced on an Illumina NovaSeq 6000 using 150 bp paired‐end reads.

Raw CUT&Tag reads were trimmed and filtered for quality control using Trim Galore v0.6.7. Clean paired‐end reads were first mapped to mouse genome mm10 using Bowtie2 (v2.4.5) with parameters ‘–very‐sensitive ‐X 2000’. PCR duplicates were removed with sambamba (V0.6.8). Only concordantly mapped reads were used for further analyzing. For histone marks enriched in Narrow domains, the NarrowPeak representation was used applying following settings: ‐f BAM –keepdup all ‐q 0.05 –bdg. For histone marks enriched in broad domains, the broadPeak representation was used applying following settings: ‐f BAM –keepdup all ‐q 0.05 –broad –broad‐cutoff 0.01. bigwig files required for coverage tracks as well as heatmaps were generated using Deeptools v3.3.1.

### Identification of Cell Cycle through Image Analysis

To remove the background illumination using Cellprofiler (v4.2.5), the images were divided into multiple blocks and found the minimum pixel intensities in blocks across the image. Then, the intensity was fitted to a polynomial and applied subtraction. To subtract the background signal, the illumination‐corrected images were processed using the rolling ball background subtraction algorithm in ImageJ.

Both nuclear and H3K4me3/H3K27me3 channel segmentation and measurements were performed in CellProfiler (v4.2.5). We used the ‘cyto2’ model from cellpose for cell segmentation and measured the fluorescence intensity and cell morphology for DAPI and IF channels, respectively.

The specified parameters were normalized using Min‐Max normalization, and then dimensionality reduction was performed using ‘DDRTree’ package with one reduced dimension and two nodes allowed in the regularization graph. The pseudotime distribution plot of the cell cycle was created with the integrated DNA fluorescence intensity on the y‐axis and the DDRTree dimensionality reduction data on the x‐axis.

### Quantitative PCR

Briefly, total RNAs were extracted by chloroform‐isopropanol method. The first‐strand cDNAs were synthesized with ReverTra Ace (Toyobo, Cat# 7601009), oligo‐dT (Takara, Cat# 3806), and RRI (Takara, Cat# 2313A). qRT‐PCR was performed on a CFX96 real‐time system (Bio‐Rad) with ChamQ SYBR qPCR Master Mix (Vazyme, Cat# Q311).

### Dual‐Luciferase Reporter Assay

6 × OCT4 or 6 × SOX2 motif was cloned into pGL3‐Basic vector (Promega, E1751). 293T cells were seeded into 24‐well plates at a density of 5 × 10⁴ cells per well and cultured overnight in DMEM supplemented with 10% FBS at 37 °C with 5% CO₂. Cells were transfected using Lipofectamine 3000 (Invitrogen) with pGL3 firefly luciferase reporter vector containing OCT4 or SOX2 binding sites, pRL‐CMV‐Renilla luciferase control plasmid, and the corresponding transcription factor expression plasmid (OCT4, OCT4‐VP16, SOX2, SOX2‐VP16, or FLAG). Cells were incubated for 48h post‐transfection before harvesting. Cell lysis and luciferase measurements were conducted using the Dual‐Luciferase® Reporter Assay System (Promega) according to the manufacturer's instructions. Transcription factors’ firefly luciferase activity was normalized to Renilla luciferase activity, and then compared with FLAG to calculate their activities.

### Bisulfite Sequencing

Genomic DNA was extracted from either OSK or OvSvK iPS cell lines using the Wizarad® Genomic DNA Purification Kit (Promega) following the manufacturer's instructions. 1 µg gDNA was used as input to bisulfite conversion using the EpiArt Ultrafast Magnetic DNA Methylation Bisulfite Kit (Vazyme, Cat# EM113) according to the manufacturer's protocol. PCR primers were synthesized according to previously published paper.^[^ ^46^ ^]^ The PCR reaction was carried out using 2 × EpiArt HS Taq Master Mix (Vazyme, Cat# EM201) following the manufacturer's instructions. PCR products were purified and cloned into the pMD18‐T Vector (TaKaRa) for transformation into DH5α competent cells. Clones were selected by PCR were used to sequencing. Data were analyzed by QUMA (http://quma.cdb.riken.jp).

### Statistical Analysis

Data were analyzed and compared by Student's t‐tests, one‐way ANOVA with Dunnett's test as a post hoc test, or two‐way ANOVA with Bonferroni's test as a post hoc test with GraphPad Prism 8.0. Error bars represent standard deviations or standard error as indicated, and “n” represents the number of independent experiments. “*”, “**”, and “***” represent significant differences (*p* < 0.05), (*p* < 0.01), and (*p* < 0.001) from indicated control groups, respectively. Statistical parameters including statistical analysis, statistical significance, and “n” value are reported in the Figure legends and Supporting Information Figure legends.

## Conflict of Interest

The authors declare no conflict of interest.

## Author Contributions

H.Z., J.K.C., and D.Q.P. were responsible for the conceptualization and supervision of the study. Data curation and formal analysis were carried out by L.G., H.Z., H.S., and H.F.G. The investigation was conducted by L.G., J.C.L., Q.W.R., H.S., Y.H.W., H.F.G., X.L.W., L.H.L., L.N.L., C.P.L., H.L., Y.B.M., S.L.C., J.D.L., and J.L. The original draft of the manuscript was written by L.G. and H.Z. Writing—Review and Editing was also performed by L.G. and H.Z.

## Supporting information



Supporting Information

## Data Availability

The data that support the findings of this study are available in the supplementary material of this article.
