# Supplementary material for: Enhanced Activities of OCT4 and SOX2 Promote Epigenetic Reprogramming by Shortening G1 Phase
Source: Adv Sci (Weinh). 2025 May 31;12(32):e15528. doi: 10.1002/advs.202415528 (PMC12407333; doi:10.1002/advs.202415528)
Supplement: Supplementary file 1 — Supporting Information [file ADVS-12-e15528-s001.docx]

Supporting Information

**Enhanced Activities of OCT4 and SOX2 Promote Epigenetic Reprogramming by Shortening G1 Phase**

*Lin Guo*, Jiechun Lin, Qiwen Ren, Hao Sun, Yanhua Wu, Haofei Ge, Xiaolan Wu, Lihui Lin, Lining Liang, Changpeng Li, He Liu, Yuangbang Mai, Shilong Chu, Jiadong Liu, Jing Liu, Jiekai Chen, Duanqing Pei*, Hui Zheng**


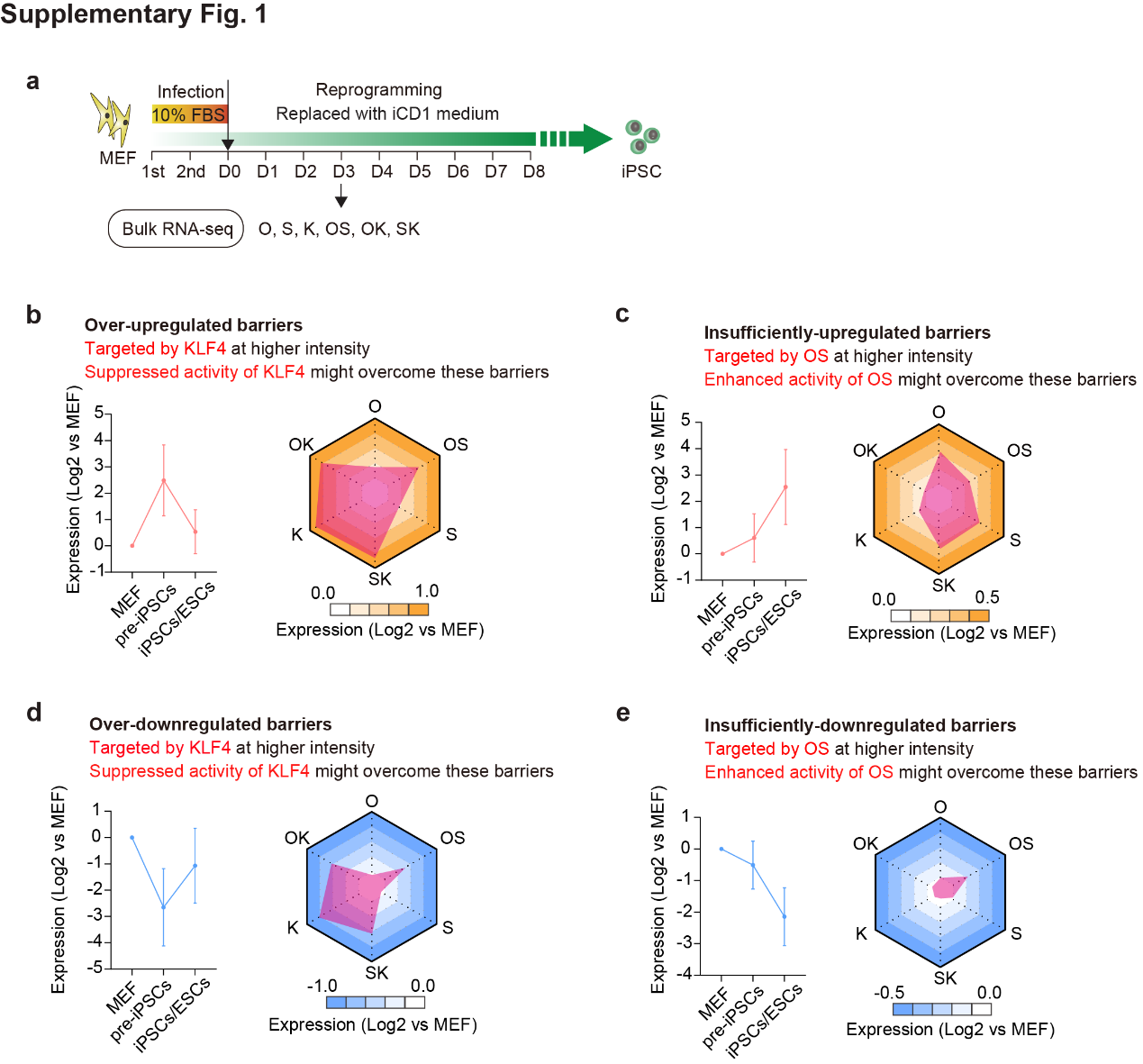


**Figure S1 KLF4 induces more reprogramming barriers than OCT4 and SOX2. a** Bulk RNA-seq was performed on day 3 during reprogramming with O, S, K, OS, OK, or SK. The corresponding results were used to identify the downstream targets of O, S, K, OS, OK, or SK. **b-e** Potential reprogramming barriers were identified by comparing the expression profiles of MEFs, pre-iPSCs, iPSCs, and ESCs (GSE14012 and GSE10871). The over up and downregulated barriers were genes with higher (over twofold) expression in pre-iPSCs than in MEFs and iPSCs/ESCs. The not sufficiently up and downregulated barriers were genes with higher (over twofold) expression in iPSCs/ESCs than in MEFs or pre-iPSCs. The abilities of O, S, K, OS, OK, and SK to regulate the expression of these four groups of barriers were summarized based on the RNA-seq results in **a**. KLF4 contributed more to reprogramming barriers than OCT4 and SOX2.


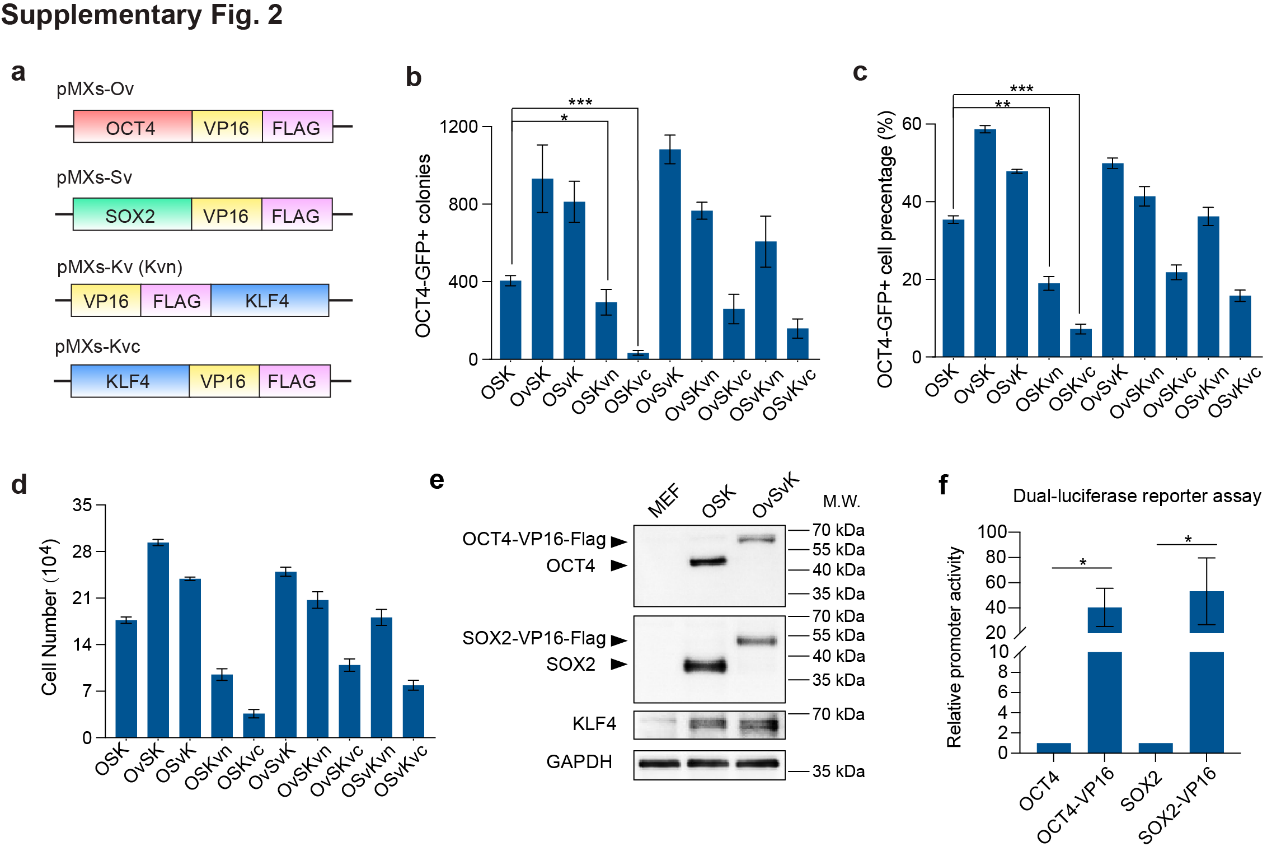


**Figure S2 OvSvK promotes somatic cell reprogramming. a** Vector construction strategy of Ov, Sv, Kvn and Kvc. **b-d** OCT4-GFP^+^ colonies, OCT4-GFP+ cell percentage, and cell proliferation were detected at day5 post-infection (n=4, mean±SD), **P* < 0.05; ***P* < 0.01; ****P* < 0.001, Student’s *t*-test. **e** Western blot analysis of OCT4, OCT4-VP16, SOX2, SOX2-VP16 and KLF4 in OSK and OvSvK systems. **f** Dual-luciferase reporter assay to assess the activity of OCT4, OCT4-VP16, SOX2, and SOX2-VP16 (n=2, mean±SD), **P* < 0.05, Student’s *t*-test.


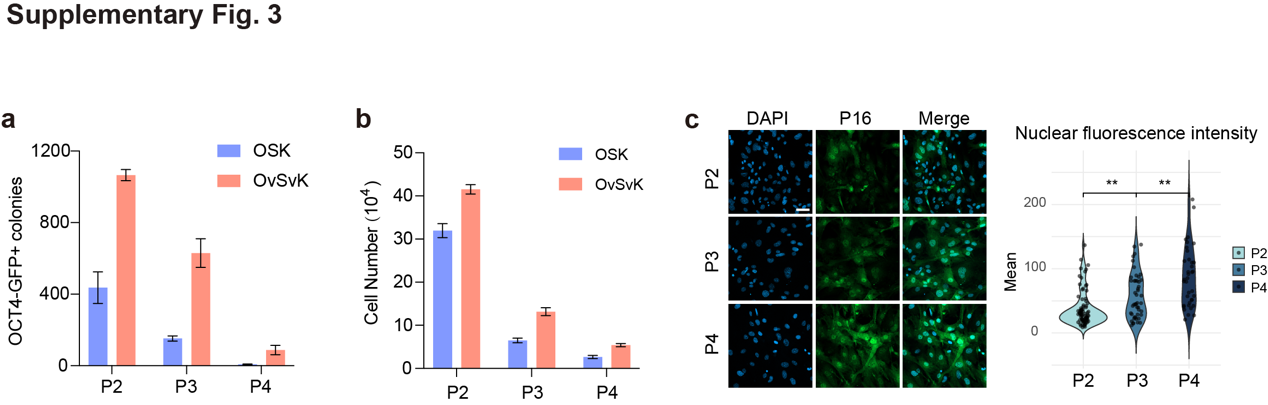


**Figure S3 OvSvK reprogramming efficiency in senescent cells. a** Comparison of reprogramming efficiency of MEF cells at different passage numbers under OSK and OvSvK conditions. OCT4-GFP^+^ colonies were counted at day 5 post-infection (n=3, mean±SD). **b** Cell proliferation analysis under different conditions at day 5 post-infection (n=3, mean±SD). **c** Immunofluorescence analysis of P16 expression in MEF cells at different passages (left panel; scale bar: 50 μm). Nuclear fluorescence intensity analysis of MEF cells at different passages by ImageJ.


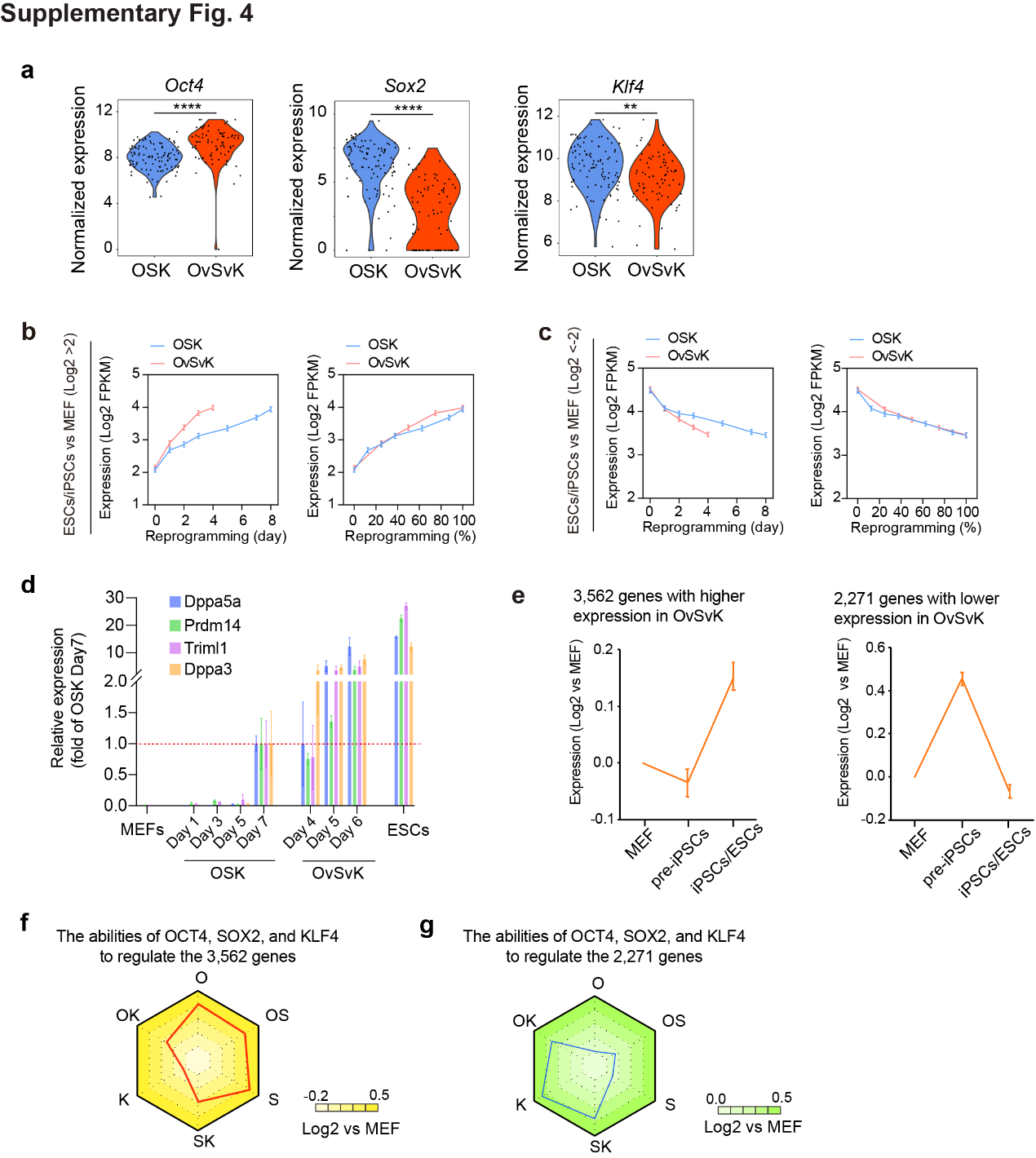


**Figure S4 Comparison of reprogramming progression between OSK and OvSvK**. **a** Expression levels of O/Ov, S/Sv, and KLF4 in scRNA-seq analysis of OSK and OvSvK reprogramming at day 1. **b** Expression trends of ESC/iPSC highly expressed genes under OSK and OvSvK reprogramming conditions over real-time progression (left) and pseudotime progression (right). **c** Expression trends of MEF highly expressed genes under OSK and OvSvK reprogramming conditions over real-time progression (left) and pseudotime progression (right). **d** qRT-PCR showed pluripotent gene expression levels at different time points (n=3, mean±SD). **e** Comparison of the two reprogramming systems revealed 3,562 genes (set 1) with higher expression and 2,271 genes (set 2) with lower expression in the OvSvK system. The expression of these two sets of genes was analyzed in MEFs, pre-iPSCs, iPSCs and ESCs (GSE14012 and GSE10871). Most of these differences were beneficial for cells to overcome the reprogramming barriers mentioned above. **f-g** The abilities of OCT4 and SOX2 to regulate the expression of these two sets of genes in Figure S4e during somatic cell reprograming.


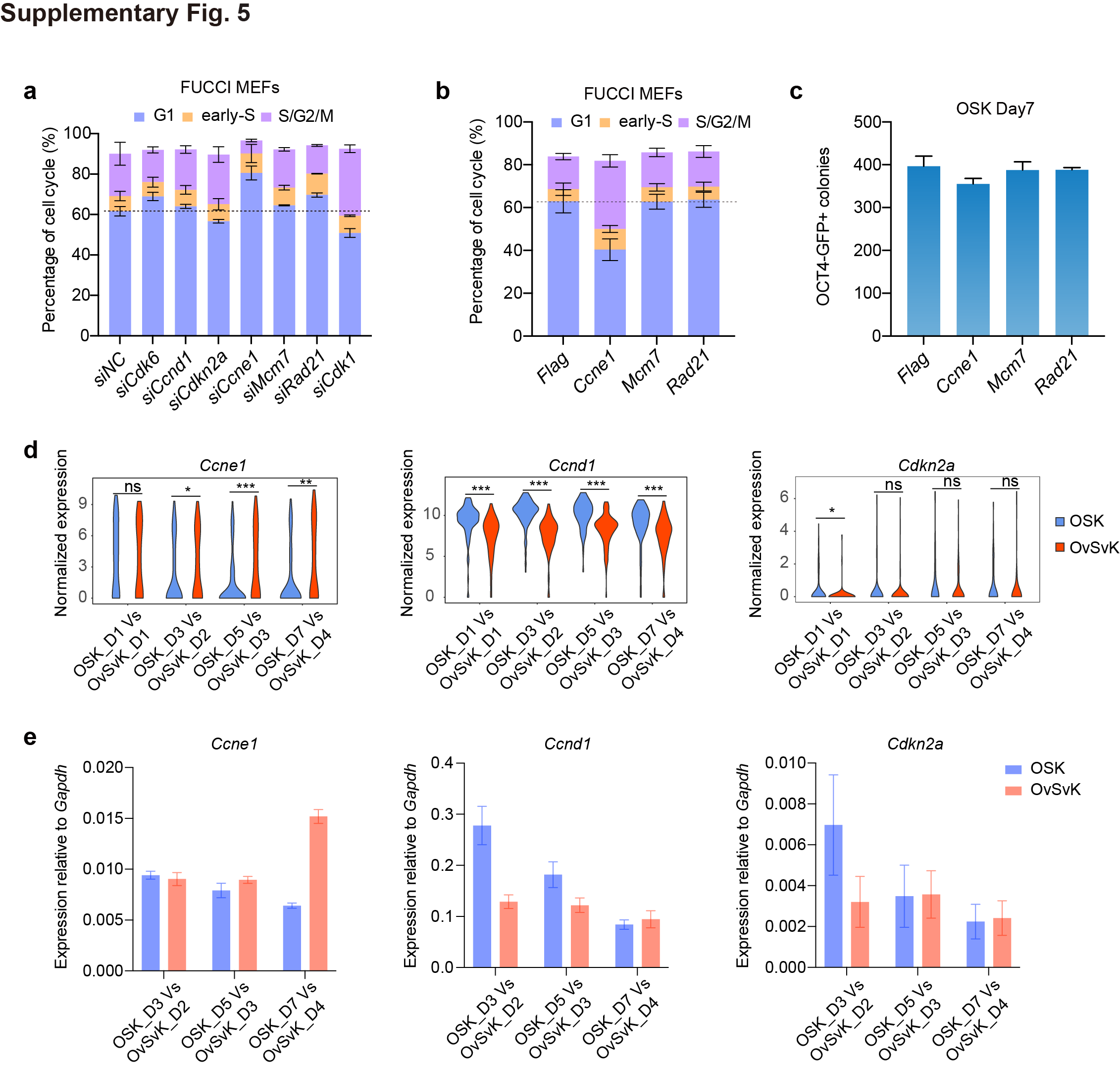


**Figure S5 Cell cycle-related genes accelerated reprogramming. a** Indicated siRNAs were transfected into FUCCI MEFs and cell cycle characteristics were assessed using FACS on day 1. **b** Indicated genes were overexpressed in FUCCI MEFs and cell cycle characteristics were analyzed using FACS on day 1. **c** OCT4-GFP^+^ colonies were analyzed on day 7 during OSK reprogramming with over-expression of different genes. **d** Expression levels of *Ccne1*, *Ccnd1* and *Cdkn2a* in scRNA-seq analysis. **e** qRT-PCR showed the expression levels of *Ccne1*, *Ccnd1* and *Cdkn2a* at different time points (n=3, mean±SD).


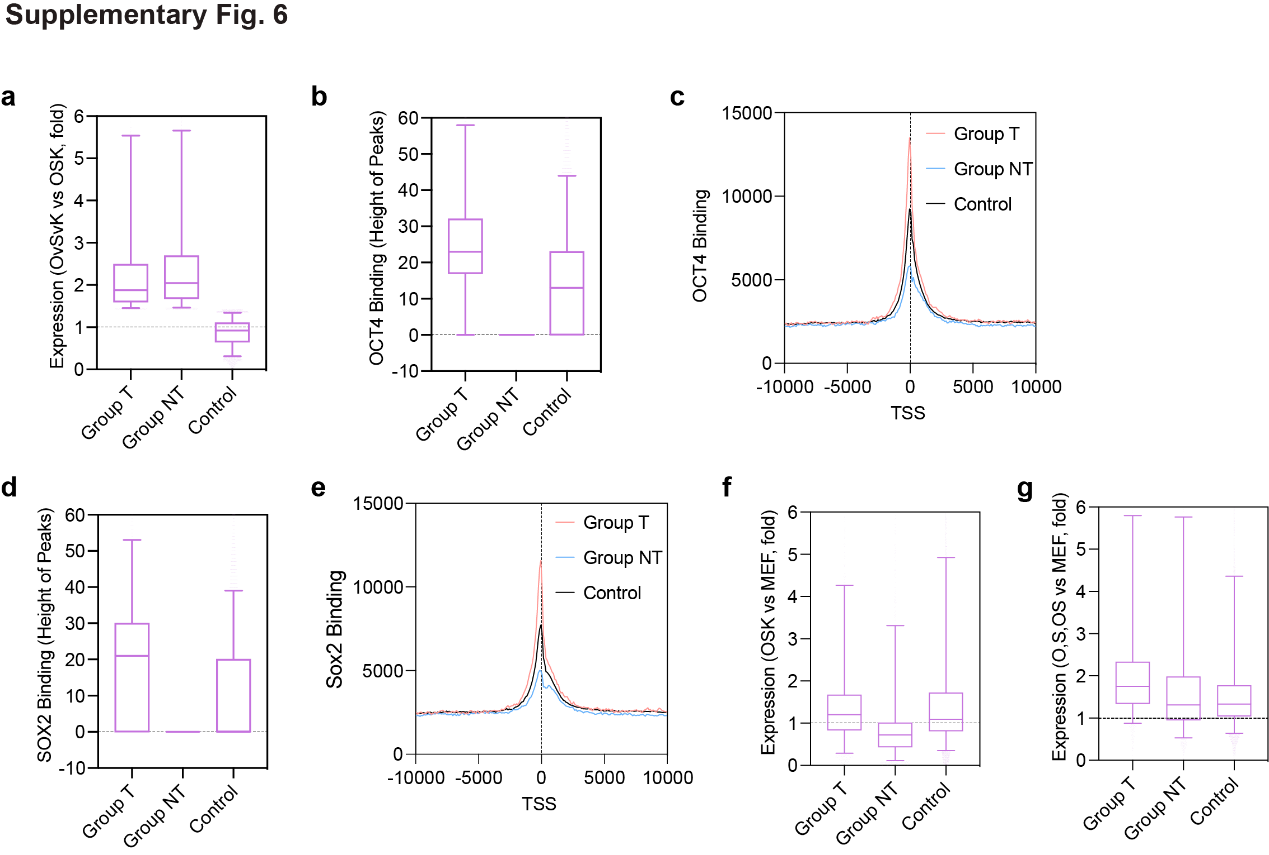


**Figure S6 The expression of genes in Group T and Group NT during reprogramming.** **a** The 15,087 genes identified in the current scRNA-seq were further divided into three groups. Genes in Group T and Group NT had higher expression on day 2 during OvSvK reprogramming than those during OSK reprogramming. **b** OCT4 binding peaks in Group T were higher than Group NT. **c** Comparison of Group T and Group NT regarding the average profile of OCT4 ChIP peaks binding to the TSS region showed that OCT4 exhibited a higher binding ability to genes in Group T. **d** Sox2 binding peaks in Group T were higher than in Group NT. **e** Comparison of Group T and Group NT regarding the average profile of Sox2 ChIP peaks binding to the TSS region showed that Sox2 exhibited a higher binding ability to genes in Group T. **f-g** Genes in Group NT were neither activated on day 2 during OSK reprogramming (**f**) nor on day 3 during reprogramming with OCT4, SOX2, or OCT4+SOX2 (**g**).


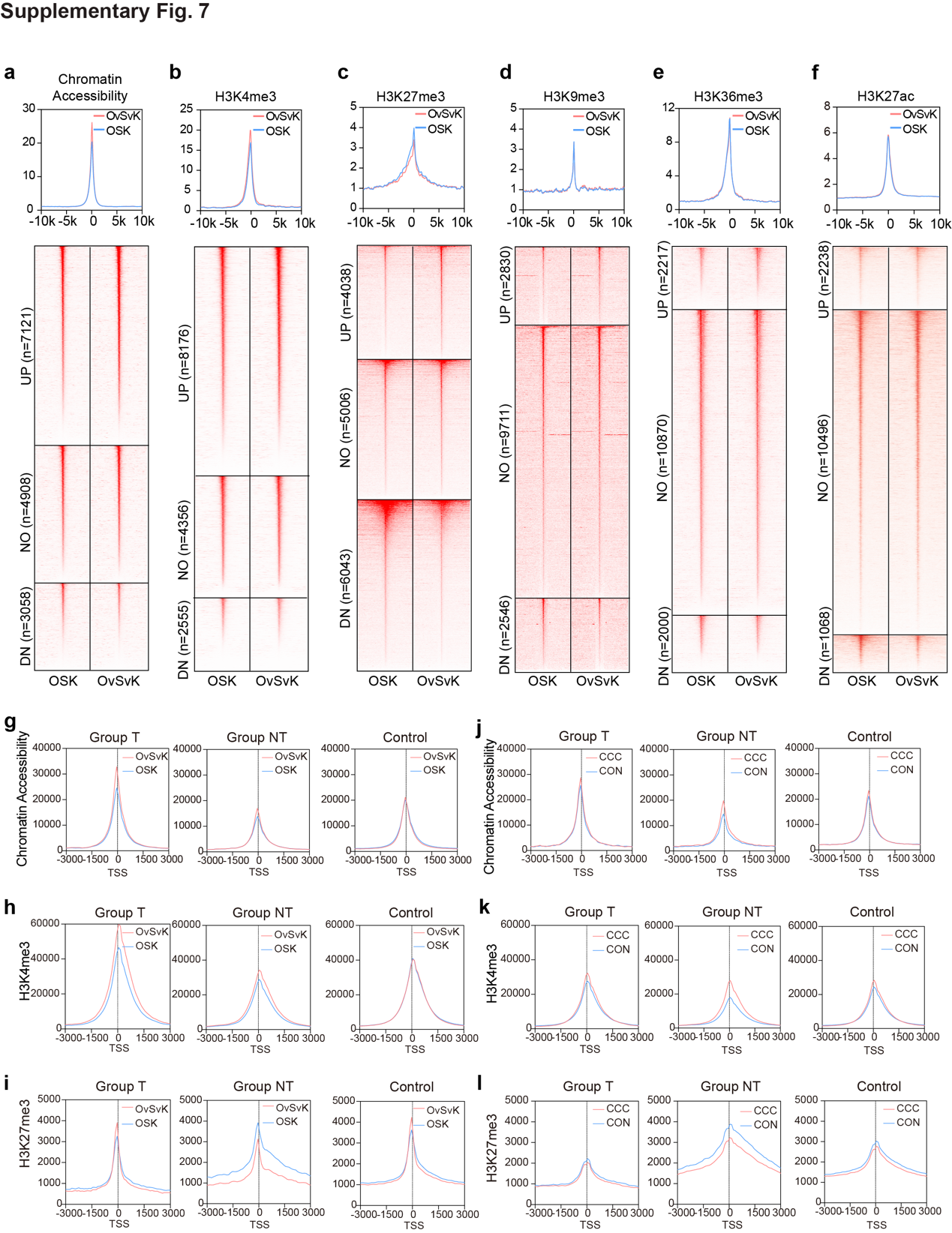


**Figure S7** **Epigenetic landscape during OSK and OvSvK reprogramming.** **a-f** Heatmap of ATAC-seq (**a**), H3K4me3 (**b**), H3K27me3 (**c**), H3K9me3 (**d**), H3K36me3 (**e**) and H3K27ac (**f**) signals of cells during OSK and OvSvK reprogramming. UP genes had higher levels of epigenetic modification in OvSvK system, while DN genes had lower. NO genes had similar levels of epigenetic modification in OSK and OvSvK systems. **g-l** Changes during reprogramming were associated with changes in chromatin accessibility and enrichment of H3K4me3 and H3K27me3 around the transcription start site (TSS) for three groups of genes, comparing OvSvK to OSK (**g-i**) and CCC to CON (**j-l**).


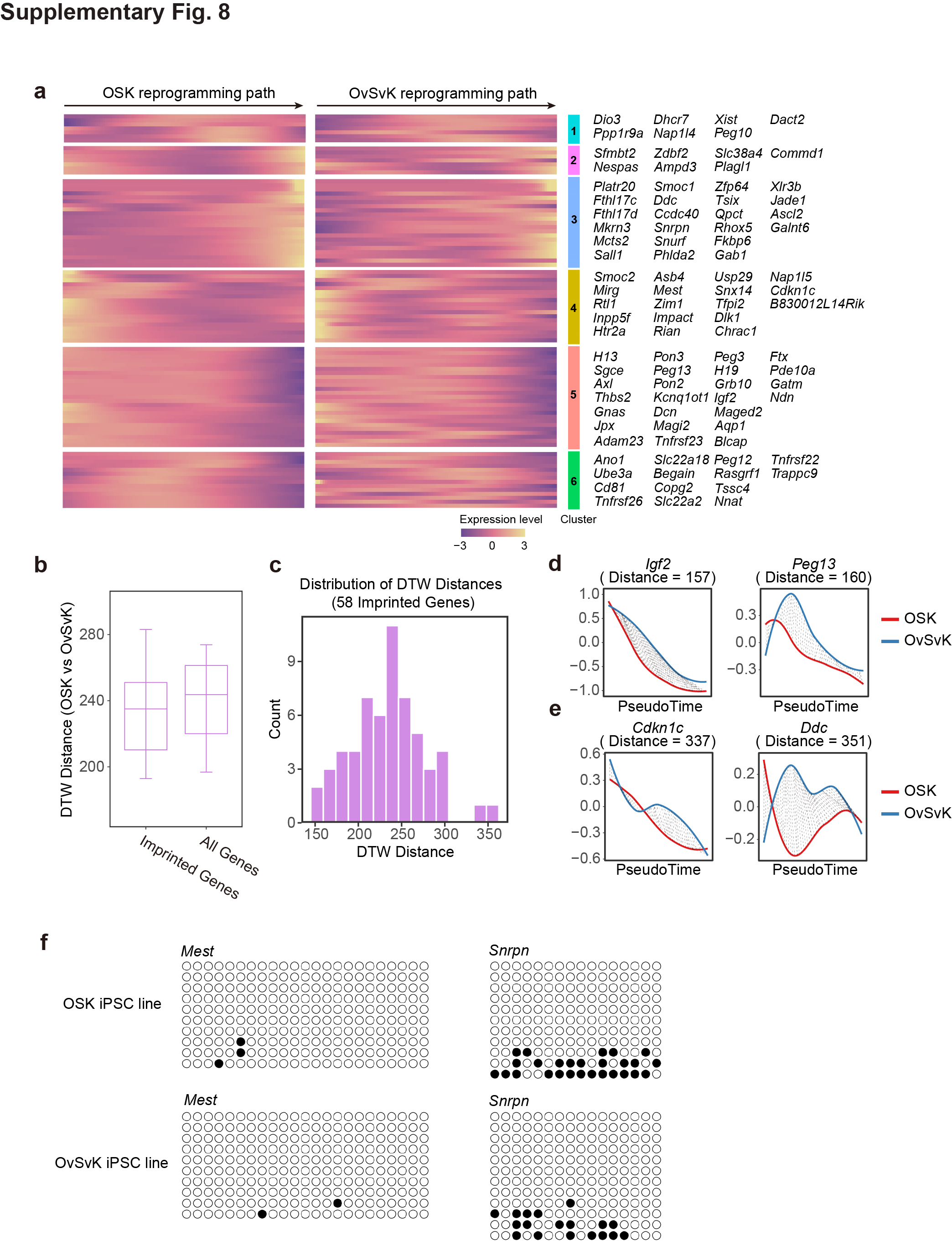


**Figure S8** **Expression changes of imprinted genes in OSK and OvSvK.** **a** Expression changes of 143 mouse imprinted genes (https://www.geneimprint.com/) along the reprogramming trajectory in the OSK and OvSvK systems using scRNA-seq data. **b** Dynamic Time Warping (DTW) analysis of expression trends for 58 imprinted genes during OSK and OvSvK reprogramming. These imprinted genes display significantly higher similarity in expression trajectories between the two reprogramming conditions, as reflected by lower DTW distances compared to all genes. The analysis was restricted to genes expressed in ≥50 cells with normalized expression > 3 in both OSK and OvSvK conditions. **c** Frequency distribution of Dynamic Time Warping (DTW) distances among 58 imprinted genes. **d** Example of two imprinted genes with small DTW distances, illustrating high expression similarity between the OSK and OvSvK reprogramming trajectories. **e** Example of two imprinted genes with large DTW distances, indicating low expression similarity between the OSK and OvSvK reprogramming trajectories. **f** Methylation levels of *Mest* and *Snrpn* validated by bisulfite sequencing in both OSK and OvSvK iPSC cell line.


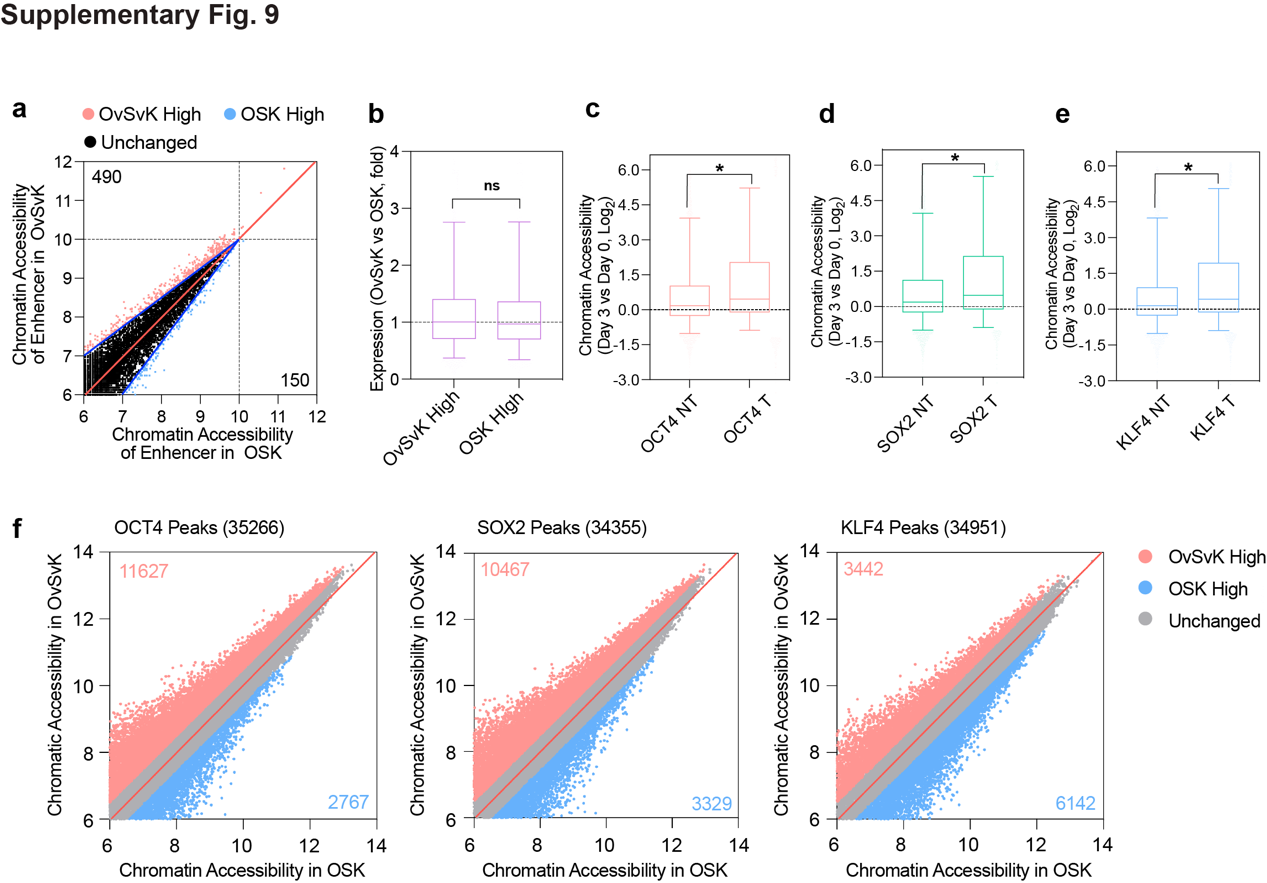


**Figure S9 Chromatin accessibility during reprogramming.** **a** Chromatin accessibility was compared in OvSvK and OSK systems. **b** There was no significant difference in gene expression between OvSvK High and OSK High groups. **c-e** The ATAC-seq dataset from GSE93029 were utilized here. Chromatin accessibility in OCT4 (**c**), Sox2 (**d**), and KLF4 (**e**) Target (T) was slightly higher than OCT4 (**c**), Sox2 (**d**), and KLF4 (**e**) Non-Target (NT). **f** Chromatin accessibility of OCT4, SOX2, and KLF4 was compared in OvSvK and OSK systems.

**
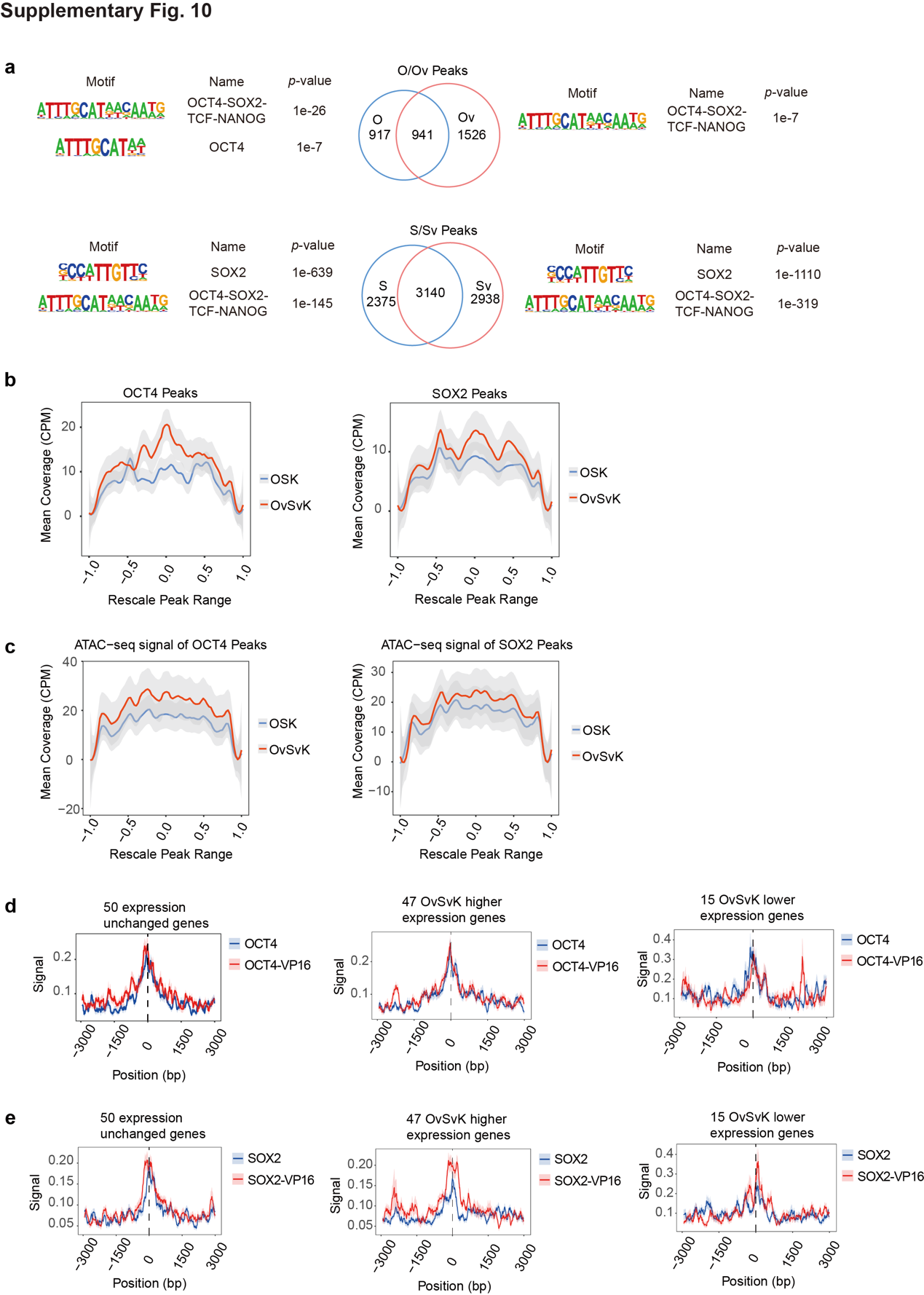
**

**Figure S10 OvSvK reprogramming enhanced OCT4 and SOX2 activity as revealed by CUT&Tag and ATAC-seq analysis. a** CUT&Tag was performed on OSK reprogramming at day 3 and OvSvK reprogramming at day 2 using Flag antibodies. The identified peaks were analyzed for motif enrichment and visualized for overlap using a Venn diagram. **b-c** The average relative coverage of peaks for OCT4 and SOX2 in CUT&Tag and ATAC-seq. OvSvK reprogramming resulted in enhanced OCT4 and SOX2 binding. **d-e** The average signal intensity at the transcription start site (TSS) region (± 3000 bp) for 50 unchanged expression genes, 47 OvSvK higher expression genes, and 15 OvSvK lower expression genes.

**
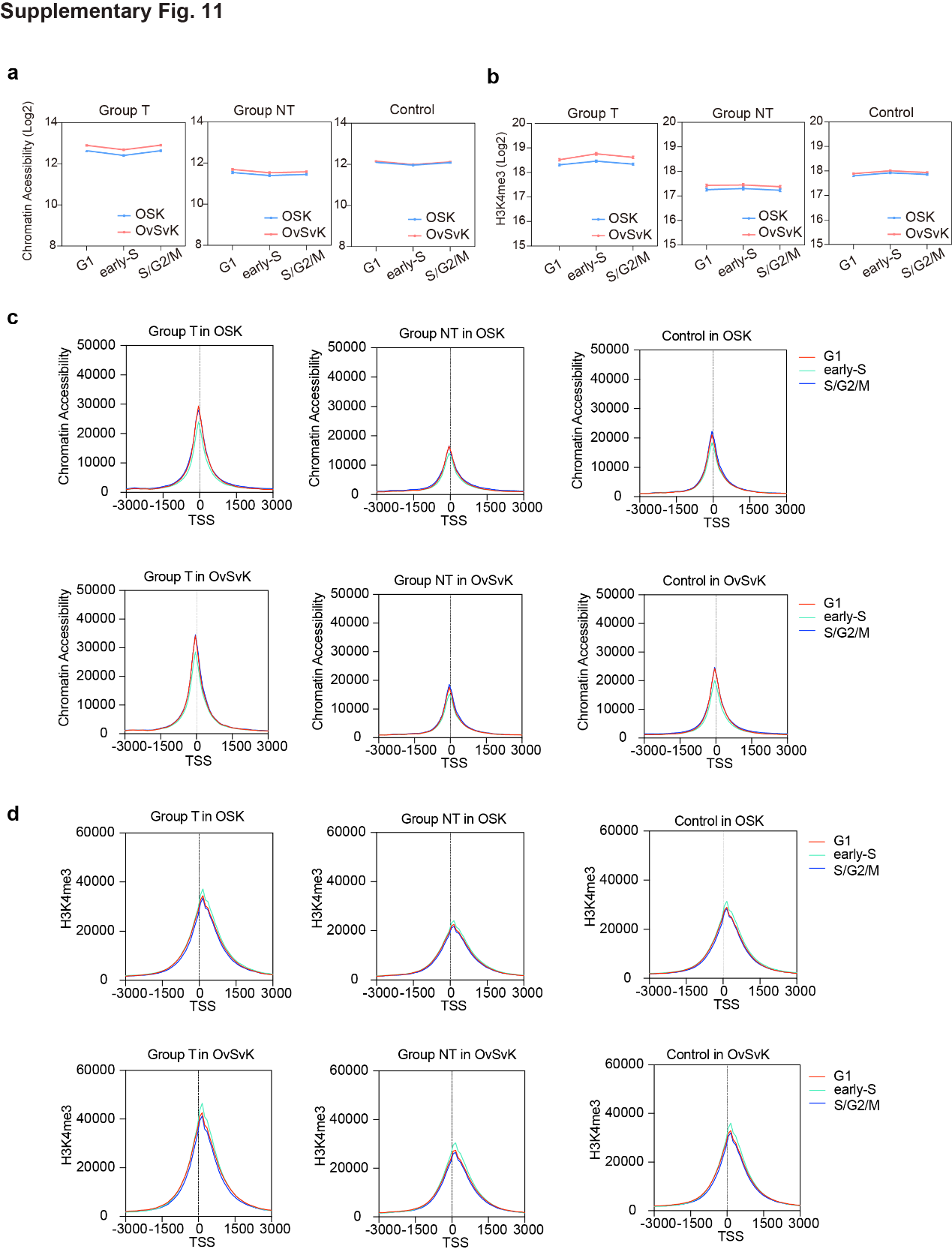
**

**Figure S11 Chromatin accessibility and H3K4me3 changes across the cell cycle. a-d** Changes in chromatin accessibility (**a,c**) and H3K4me3 enrichments (**b,d**) around the transcription start site (TSS) for three groups of genes at different cell cycle stages in OSK and OvSvK systems.

**
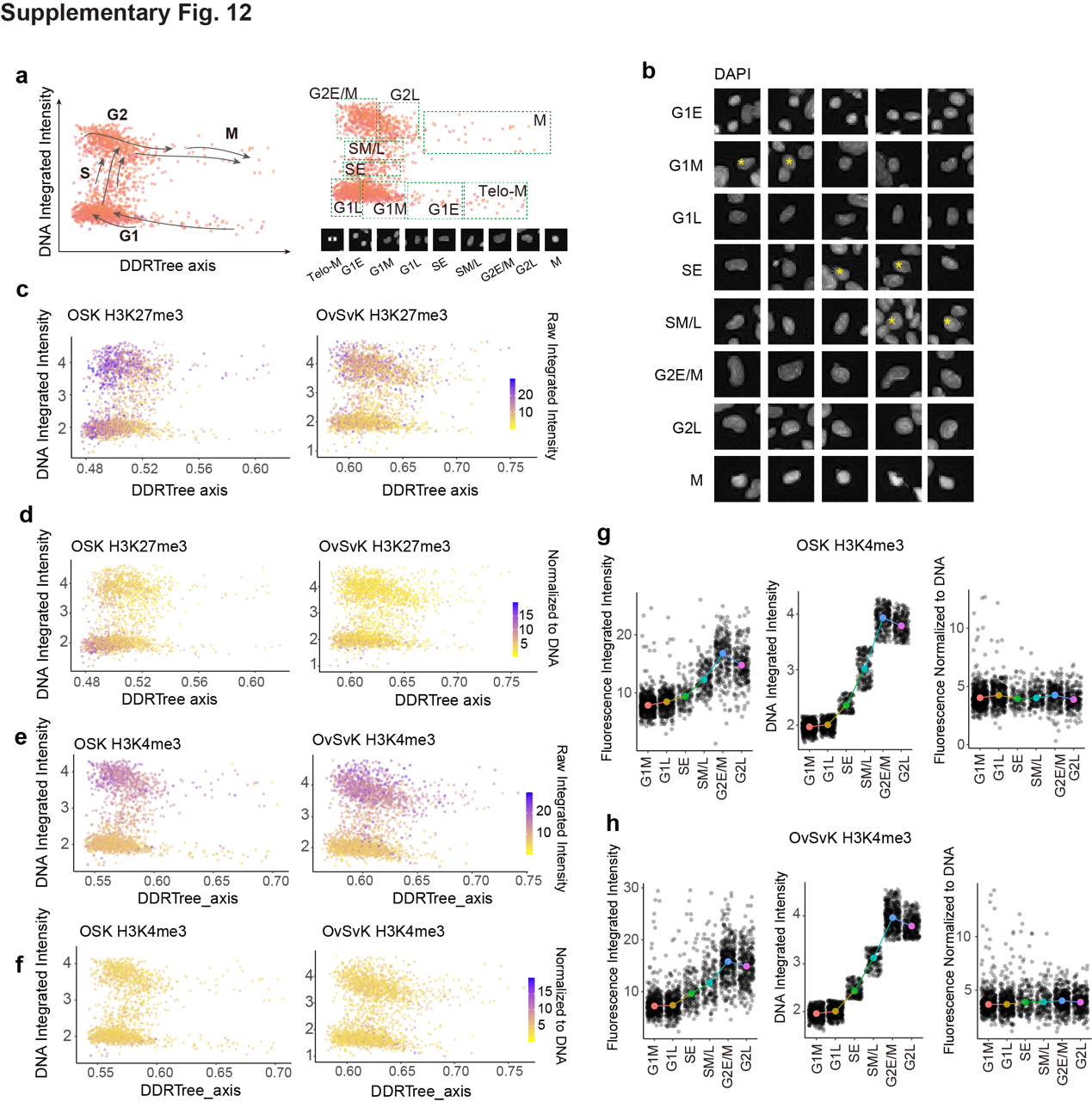
**

**Figure S12 Changes in H3K4me3 and H3K27me3 across the cell cycle in the OvSvK and OSK systems. a** Image-based cell cycle pseudotime analysis. **b** The displayed nuclei represent the cell cycle stages as defined by the cell cycle pseudotime algorithm. Asterisks (*) indicate corresponding nuclei at each cell cycle stage. **c-d** The pseudotime distribution showing the mapping of H3K27me3 integrated fluorescence intensity (**c**) and DNA-normalized relative fluorescence intensity (**d**). **e-f** The pseudotime distribution showing the mapping of H3K4me3 integrated fluorescence intensity (**e**) and DNA-normalized relative fluorescence intensity (**f**). **g-h** Statistics of integrated fluorescence intensity of H3K4me3 (**g-h**, left), integrated fluorescence intensity of DNA (**g-h**, middle), and relative modified histone fluorescence intensity normalized to DNA (**g-h**, right) across various cell cycle stages.

**
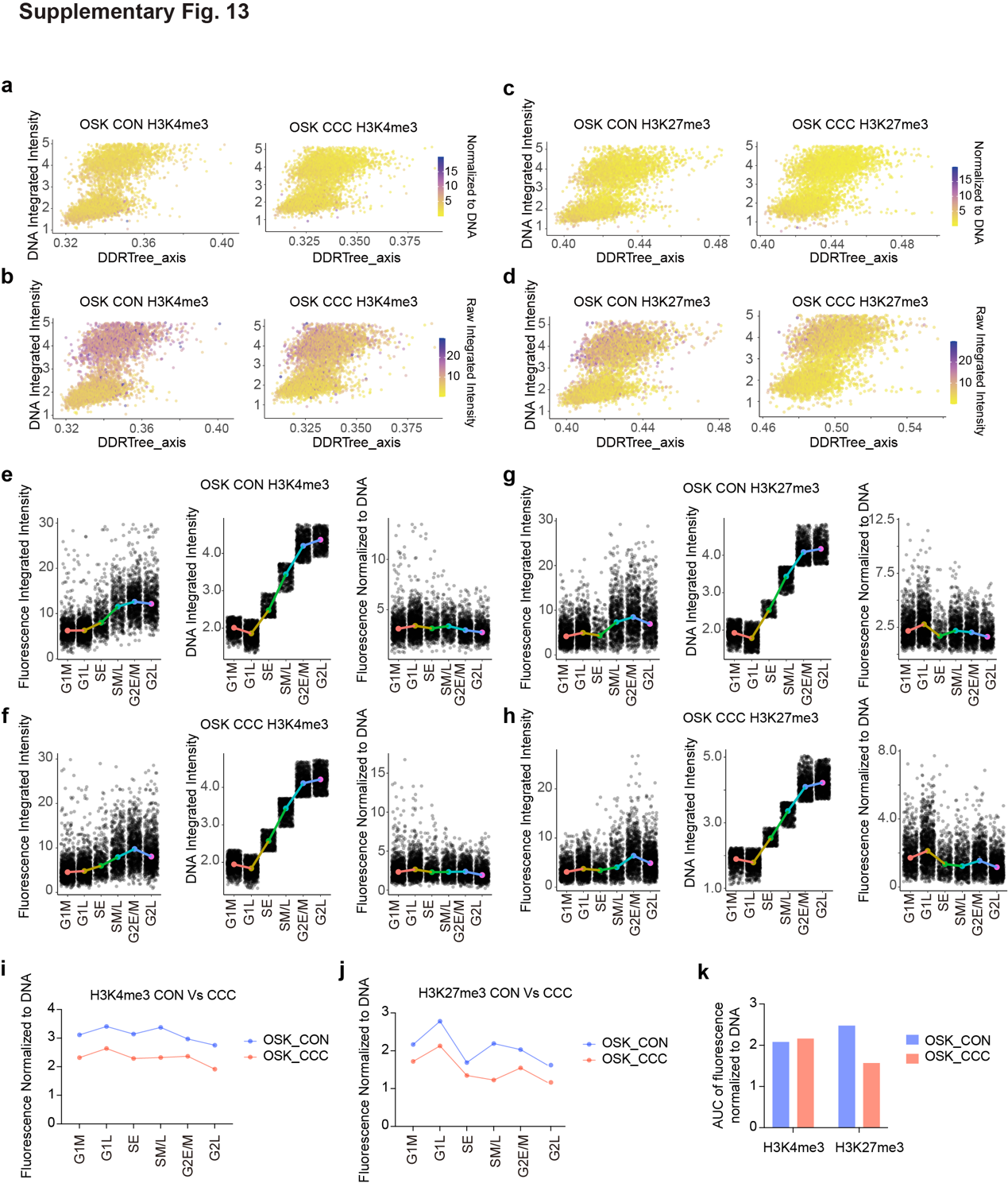
**

**Figure S13 Changes in H3K4me3 and H3K27me3 across the cell cycle in the CCC and CON systems. a-d** The pseudotime distribution showing the mapping of H3K4me3 and H3K27me3 integrated fluorescence intensity and DNA-normalized relative fluorescence intensity. **e-h** Statistics of integrated fluorescence intensity of H3K4me3 (**e-f**) and H3K27me3 (**g-h**), integrated fluorescence intensity of DNA and relative modified histone fluorescence intensity normalized to DNA across various cell cycle stages. **i-k** The DNA-normalized relative fluorescence intensity of H3K4me3 (**i**) and H3K27me3 (**j**) across the cell cycle stages and AUC analysis (**k**).
